# Catalytic, asymmetric carbon–nitrogen bond formation using metal nitrenoids: from metal–ligand complexes *via* metalloporphyrins to enzymes

**DOI:** 10.1039/d3sc04661c

**Published:** 2023-10-25

**Authors:** Alexander Fanourakis, Robert J. Phipps

**Affiliations:** a Yusuf Hamied Department of Chemistry, University of Cambridge Lensfield Road Cambridge CB2 1EW UK rjp71@cam.ac.uk

## Abstract

The introduction of nitrogen atoms into small molecules is of fundamental importance and it is vital that ever more efficient and selective methods for achieving this are developed. With this aim, the potential of nitrene chemistry has long been appreciated but its application has been constrained by the extreme reactivity of these labile species. This liability however can be attenuated by complexation with a transition metal and the resulting metal nitrenoids have unique and highly versatile reactivity which includes the amination of certain types of aliphatic C–H bonds as well as reactions with alkenes to afford aziridines. At least one new chiral centre is typically formed in these processes and the development of catalysts to exert control over enantioselectivity in nitrenoid-mediated amination has become a growing area of research, particularly over the past two decades. Compared with some synthetic methods, metal nitrenoid chemistry is notable in that chemists can draw from a diverse array of metals and catalysts , ranging from metal–ligand complexes, bearing a variety of ligand types, *via* bio-inspired metalloporphyrins, all the way through to, very recently, engineered enzymes themselves. In the latter category in particular, rapid progress is being made, the rate of which suggests that this approach may be instrumental in addressing some of the outstanding challenges in the field. This review covers key developments and strategies that have shaped the field, in addition to the latest advances, up until September 2023.

## Abstract 2

This review discusses the varied catalyst design strategies that have been applied to asymmetric C**−**N bond formation using metal nitrenoids. The outstanding challenges are also identified with the aim of encouraging further investigation in the field.

## Graphical abstract

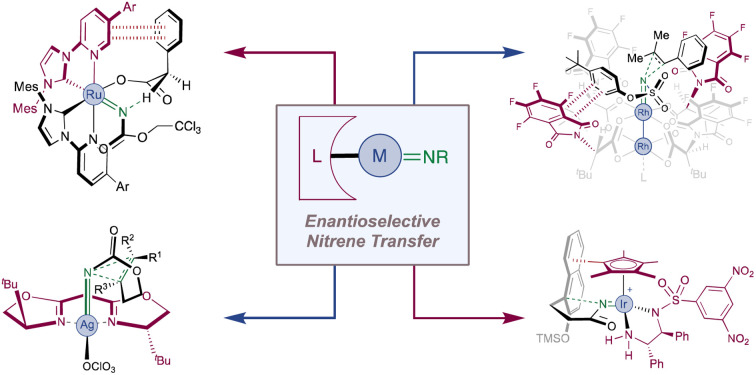

## 1. Introduction

The synthesis of aliphatic amines is central to organic chemistry and traditional disconnections such as reductive amination and alkylation have recently been complemented by new C–N bond-forming methods which can introduce nitrogen from less reactive C(sp^3^)–H bonds or alkenes.^1,2^ The development of new transition metal-catalysed processes in particular has been instrumental in accelerating the advance of the field. From these methods, functionalisation using metal nitrenoids is appealing since it provides a particularly direct approach to the formation of carbon–nitrogen bonds.^3^

**Fig. 1:**
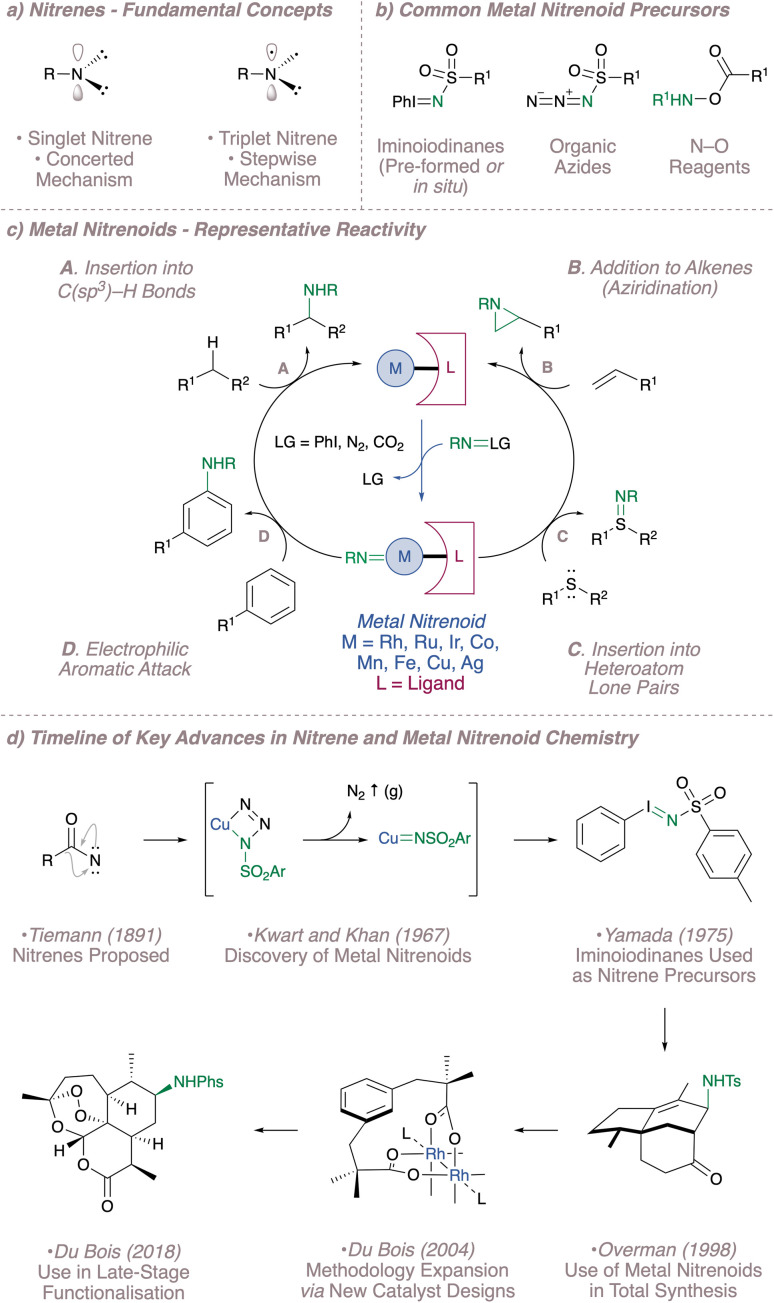
A brief summary of the development of nitrene and metal nitrenoid chemistry from 1891 to the present day.

Nitrenes themselves were already invoked as reactive intermediates as early as the late 1800s in the Lossen^4^ and Curtius^5^ rearrangements and their extreme electrophilicity is reflected in their characteristic reactivity.^6–8^ These univalent nitrogen sources, which exist in either singlet or triplet spin states, possess only six valence electrons and seek to gain the remaining pair through insertion at electron-rich sites (Fig. 1a). This encompasses insertion into aliphatic C–H bonds, addition to alkenes and aromatic π-systems and insertion into heteroatom lone pairs (Fig. 1c).^9^ These reactions are potentially useful but given the high reactivity of nitrenes, effective control over these fleeting intermediates is essential for the development of any practical process. Fortunately, free nitrenes can be tamed through association with a transition metal and a supporting secondary ligand framework to form a more discriminating reactive species.^1^

The start of modern metal nitrenoid chemistry can arguably be traced to Kwart and Khan's two consecutive publications from 1967 in which they demonstrated that copper was effective in decomposing benzenesulfonyl azide to afford a copper nitrenoid.^10,11^ The inspiration for this approach was based on the well-precedented, copper-catalysed decomposition of diazo compounds to form carbenes and indeed it could be said that developments in nitrenoid chemistry have typically lagged somewhat behind the corresponding carbenoid methodology.^12–14^ In this particular system, the products observed following reaction in the presence of DMSO or alkanes and alkenes all suggested the intermediacy of a reactive nitrenoid. Since this seminal report, metal nitrenoid chemistry has progressed from strength to strength on the one hand thanks to the continued introduction of new catalysts and on the other thanks to the introduction of new precursors to ensure metal nitrenoid generation under mild conditions (Fig. 1b and d).^15^ A timeline of selected important advances in the field is shown in Fig. 1d. In addition to the developments discussed above, these encompass the popularisation of iminoiodinanes as nitrene precursors (both pre-formed^16^ and generated *in situ*^17,18^), the early applications of intermolecular nitrenoid chemistry to complex molecule synthesis,^19–21^ the expansion of the methodology through rational catalyst design^22,23^ and most recently the advancement of the field to a stage where it can be reliably used for the latestage functionalisation of complex molecules.^24^

Many of the reactions undergone by metal nitrenoids generate at least one new stereocentre and, given the fundamental importance of enantioenriched amines in society,^25^ efforts to induce asymmetry in this chemistry are constantly being pursued. This review article will explore the strategies that have been used to render metal nitrenoid insertion into C(sp^3^)–H bonds and alkenes asymmetric. For most, an early pioneering example is briefly discussed for context, followed by an extended examination of the more recent methodologies. There have been a number of relevant reviews published previously including those that focused on aziridination^26,27^ or C–H amination alone.^28^ The most recent is from Ju and Schomaker which provides an excellent combined overview relating to the use of synthetic catalysts up until late 2020.^9^

As mentioned previously compared with some other reaction types, metal nitrenoid chemistry is notable in that a very diverse range of metals and catalysts can facilitate it, ranging from metal-ligand complexes, bearing a variety of ligand types, all the way through to engineered enzymes at the other end of the catalysis spectrum. A key difference compared with previous reviews is that this article will cover, alongside purely synthetic catalysts subdivided by ligand class (Section 2), advances in asymmetric nitrene transfer using enzymes (Section 4). Linking these together is one of the most productive synthetic catalyst architectures for asymmetric nitrene transfer: metalloporphyrins (Section 3). The biological inspiration^29^ behind these provides an insightful connection between the synthetic and enzymatic catalysts surveyed in the adjoining sections. It is notable that even since the most recent review, which did not include enzymatic approaches, there have been a substantial number of new advances in all types and the extended format possible here allows for detailed discussion which we hope will provide the reader with a broad overview of the field. The aim of our article is not only to showcase the creative catalyst designs that have enabled such great progress in asymmetric nitrene transfer so far, but also to highlight some of the limitations of the current methods which we hope will spur further development.^30^

## 2. Synthetic catalysts

### 2.1 Chiral bis(oxazoline) (BOX) ligands

Following their introduction in 1989, oxazolines and their derivatives quickly rose to prominence as a “privileged” ligand class.^31–34^ In particular, the bis(oxazolines), reported simultaneously by Evans^35^ and Corey^36^ have found widespread use ever since as a result of their facile synthesis, modular nature and well-defined open and closed quadrants.^37^ In fact, one of the earliest examples of catalysis using BOX ligands was the asymmetric copper-catalysed aziridination of cinnamate esters reported by Evans in 1993.^38^ Evans had previously applied these ligands to enantioselective cyclopropanation^35^ and also studied racemic copper-catalysed aziridination.^39^

**Fig. 2:**
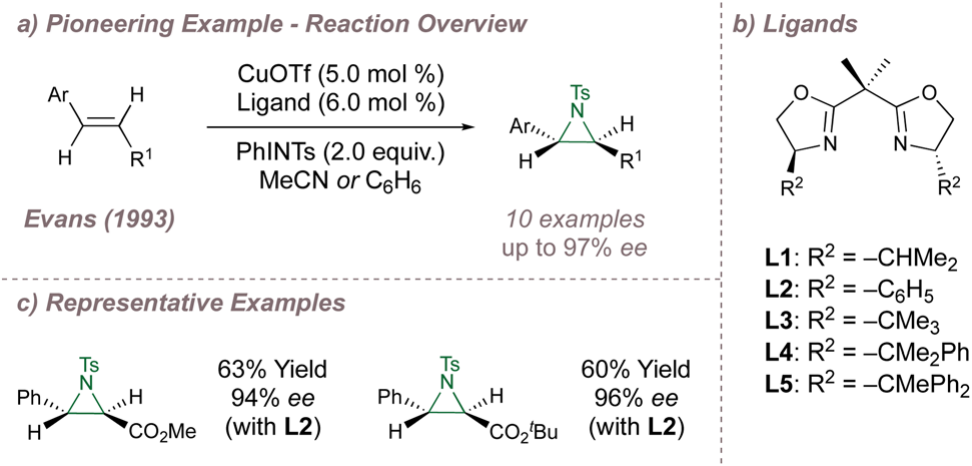
Evans' enantioselective aziridination using copper-BOX catalysis.

Systematic investigation of the copper source, solvent and steric demand of the ligand side arms (L1–L5) resulted in a highly effective aziridination protocol, delivering products in up to 97% *ee* (Fig. 2). A similar reaction using a different chiral ligand had been reported previously by Masamune^40^ although Evans raised issues concerning its reproducibility. Advances on Evans' protocol were later disclosed by Södergren who varied the electronic properties of the nitrogen source to improve reaction yields and *ee*^41,42^ and an intriguing heterogeneous system was reported by Hutchings which led to improved yields and enantioselectivities using a bis(oxazoline)-modified Cu^2+^ exchanged zeolite Y.^43,44^ During investigations to access enantioenriched α-amino ketones, Adam^45^ studied an “aza-Rubottom”^46^ aziridination-ring opening sequence of enols using a system based on Evans' report but was unable to obtain enantioselectivities greater than 52% *ee*.

**Fig. 3:**
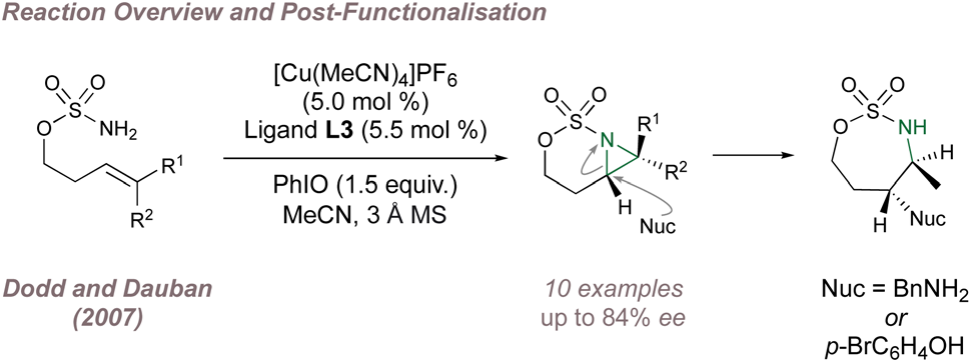
Dodd and Dauban's enantioselective intramolecular aziridination of aliphatic alkenes.

Copper-BOX complexes have also been used for asymmetric intramolecular aziridination using sulfamates and a 2007 report from Dodd and Dauban constituted the first example of intramolecular catalytic asymmetric nitrene transfer to unactivated aliphatic alkenes (Fig. 3).^47^ Varied olefin substitution was tolerated with moderate to good *ee* and the optimal substrates possessed *trans*-double bond geometry. When *cis*-alkenes were trialled, not only was the *ee* poor but traces of *trans*-aziridines were detected suggesting that a triplet nitrene intermediate was involved. The utility of the fused aziridines was demonstrated through regioselective and stereospecific ring opening using nitrogen and oxygen nucleophiles with no erosion of *ee*.

**Fig. 4:**
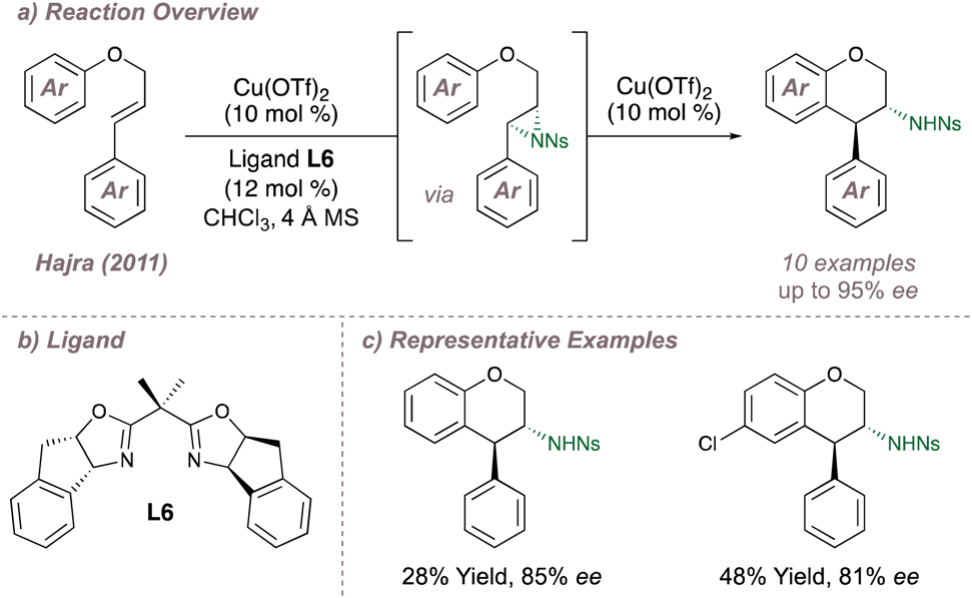
Enantioselective synthesis of *trans*-3-amino-4-arylchromans.

Continuing with intramolecular copper-catalysed aziridination reactions, in 2011 Hajra reported an enantioselective one-pot azidoarylation of aryl cinnamyl ethers (Fig. 4a).^48^ Following enantioselective aziridination at the activated alkene, an intramolecular copper-catalysed Friedel–Crafts cyclisation led to opening of the pendant aziridine with high regio- and diastereoselectivity. Up to 95% *ee* could be obtained in the products using BOX ligand L6 in conjunction with Cu(OTf)_2_ (Fig. 4b). Although the selectivities are good, the authors often obtained low to moderate product yields (Fig. 4c). This was due to competing nitrene insertion at the allylic C(sp^3^)–H bonds which in this reaction are also activated by the adjacent oxygen atom. Overall the transformation effectively builds molecular complexity and is of relevance to total synthesis given the ubiquity of the chroman motif in natural products. Hajra has reported a number of similar asymmetric aziridination/ring-opening reactions^49,50^ with a particular focus on using these as the key step in the synthesis of dopamine receptor D1 agonists and antagonists.^51–53^

**Fig. 5:**
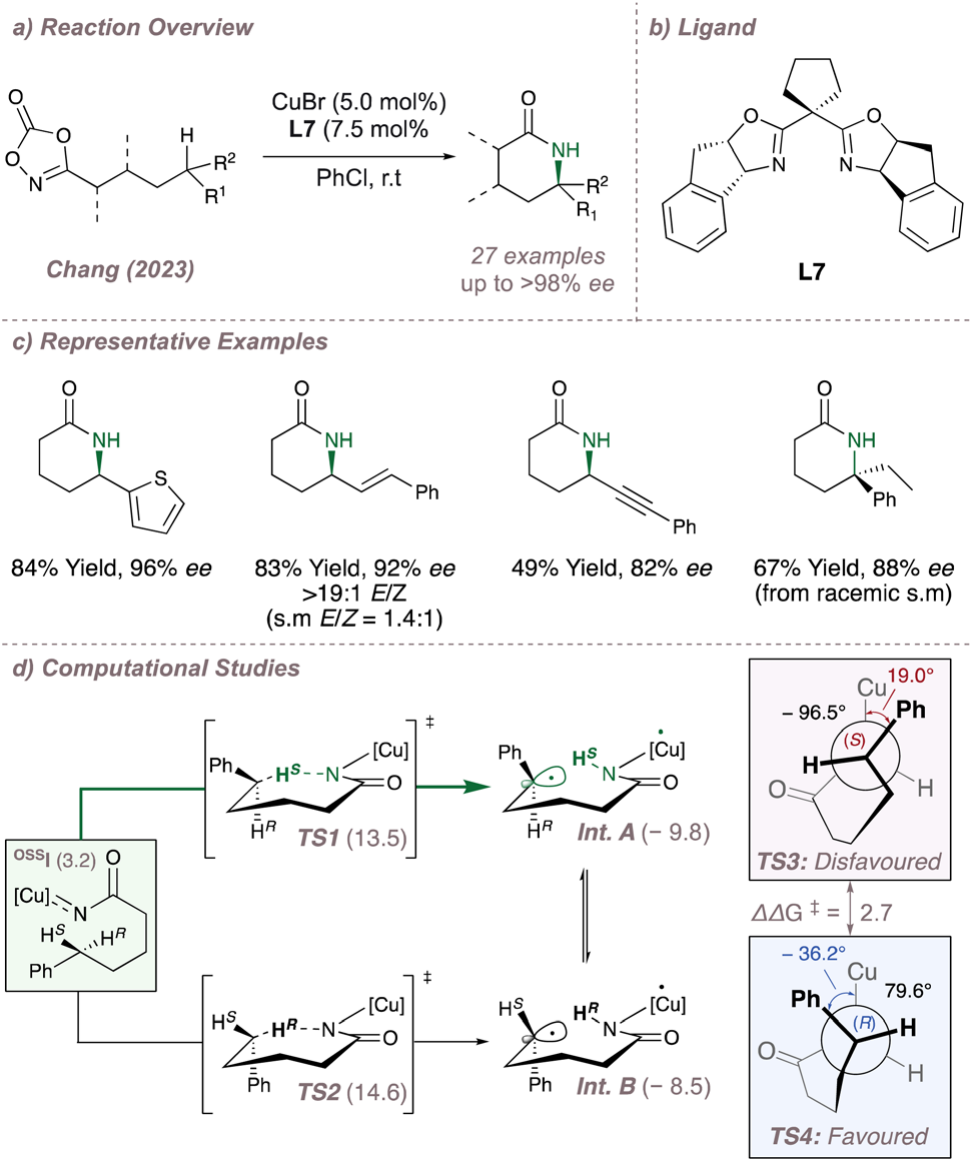
Site- and enantioselective intramolecular δ-C(sp^3^)–H amination using Cu-BOX catalysis.

Leading on from these examples, a very recent publication from Chang disclosed a Cu-BOX system for intramolecular C(sp^3^)–H amination of dioxazolones at predominantly electronically-activated positions (Fig. 5a and b).^54^ By exploiting the open-shell character of the postulated copper nitrenoids, both regio- and enantioselectivity are simultaneously controlled to afford access to versatile enantioenriched δ-lactams. Not only can these intermediates undergo further diastereoselective transformations to build up ring systems with multiple stereocentres but these also provide a useful entry point to the synthesis of several biologically active alkaloids. The δ-selectivity is unusual and as a result the valuable enantioenriched products nicely complement existing intramolecular asymmetric metalloradical cyclisations which target either β-^55^ or γ-positions (Fig. 5c). In addition to an impressive substrate scope, extensive mechanistic and computational studies were carried out to rationalise the reaction outcome (Fig. 5d). Computations suggested that the initial intramolecular hydrogen atom abstraction (HAA) occurred from an open shell singlet state ^OSS^I which was higher in energy (+3.2 kcal mol^−1^) than the corresponding triplet. The resulting 1,6-HAA (δ-selectivity) barriers were lower than the corresponding 1,5-HAA barriers (γ-selectivity) thus rationalising the regioselectivity. Considering the 1,6-HAA transition states, although TS1 is favoured over TS2 by 1.1 kcal mol^−1^, this difference is not great enough to account for the 97% *ee* observed for the model substrate and suggests that HAA is not the enantiodetermining step. Instead, it was calculated that the radical intermediates Int. A and Int. B, were long lived and could interconvert as evidenced by mechanistic studies involving the isolation of racemic, TEMPO-trapped products at the γ-position. This therefore suggested that C–N bond formation was enantiodetermining. In this Curtin–Hammett scenario, the transition state for radical rebound (TS4) (arising from the higher energy Int. B) is favoured over TS3 by 2.7 kcal mol thanks to a staggered conformation ∠Cu–N–C–C(Ph) = −36.2° which minimises unfavourable steric repulsion between the substrate and the ligand. Pleasingly, the conclusions derived from the in-depth computations were convincingly validated with experimental support. For example, when isomeric starting material mixtures such (*E*/*Z* olefins or racemates) were subjected to the reaction conditions these converged to single products with high selectivity either as a result of rapid radical alkene isomerisation or C-centred radical inversion respectively. This is in direct agreement with the proposed enantiodetermining C–N step. Other enantioconvergent radical C–N aminations (using chiral porphyrins) for both intermolecular and intramolecular functionalisation are explored in Section 3.

**Fig. 6:**
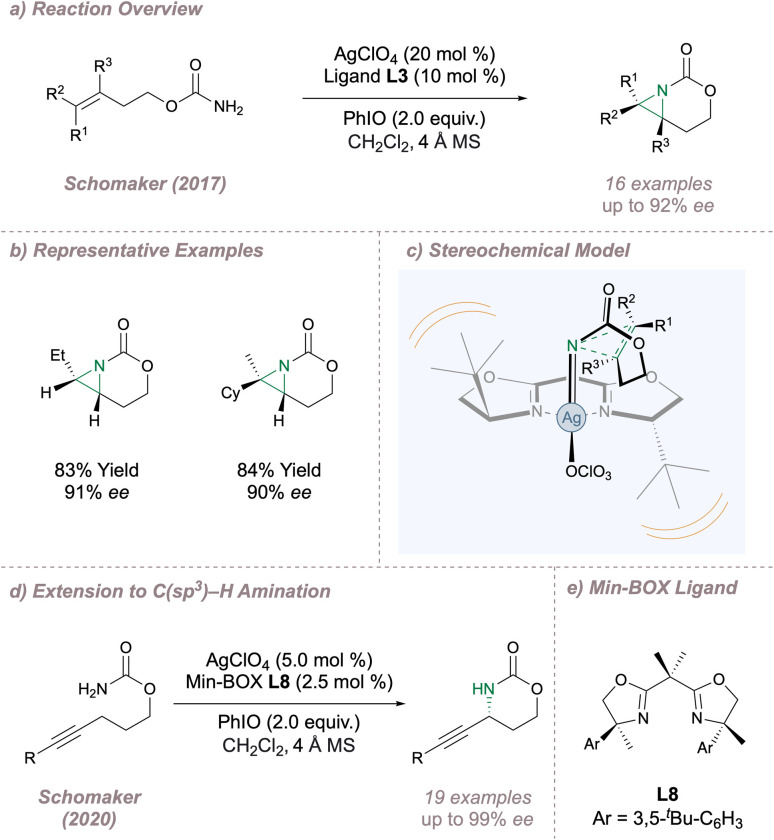
Applications of Ag(i)-BOX catalysis to asymmetric intramolecular aziridination and C(sp^3^)–H amination.

Moving to silver, the Schomaker lab has a long-standing interest in using rational catalyst design to exert control over various aspects of selectivity in silver-catalysed nitrene transfers.^56^ In 2017, this group reported a highly enantioselective intramolecular aziridination of carbamates (Fig. 6a and b) which is complementary to the protocol for sulfamates shown in in Fig. 3.^57^ During reaction optimisation the authors had to contend with competing allylic amination but this was overcome through careful choice of silver source and BOX ligand. For successful outcomes, at least one substituent was required on the distal alkene carbon (R^1^, R^2^ or both) and only monosubstitution was tolerated at the proximal site. This latter constraint arises since when R^3^ is larger than a hydrogen atom there is considerable steric clash between the substrate and the ligand backbone in the key transition state. Further, a two-carbon tether between the carbamate and the alkene was optimal. These observations formed the basis of a stereochemical model founded on the minimisation of steric clash between the substrate and ligand at the enantiodetermining transition state (Fig. 6c). As with Dodd and Dauban's fused sulfamate-derived aziridines, ring opening occurred with excellent regioselectivity, albeit now at the distal aziridine carbon, to afford valuable enantioenriched cyclic carbamates. The development of aminofunctionalisation reactions which provide similar fused aziridines remains an emerging area of significant interest.^58^ Subsequently Schomaker extended this silver-catalysed methodology to intramolecular asymmetric propargylic C(sp^3^)–H amination (Fig. 5d).^59^ Ligand optimisation resulted in the application of the Min-Box L8 containing bulky aryl side arms and full substitution α- to the nitrogen atoms (Fig. 6e). Not only does this ligand deliver the products in excellent *ee* but it also suppresses competing and undesired Ag(i) coordination modes.

**Fig. 7:**
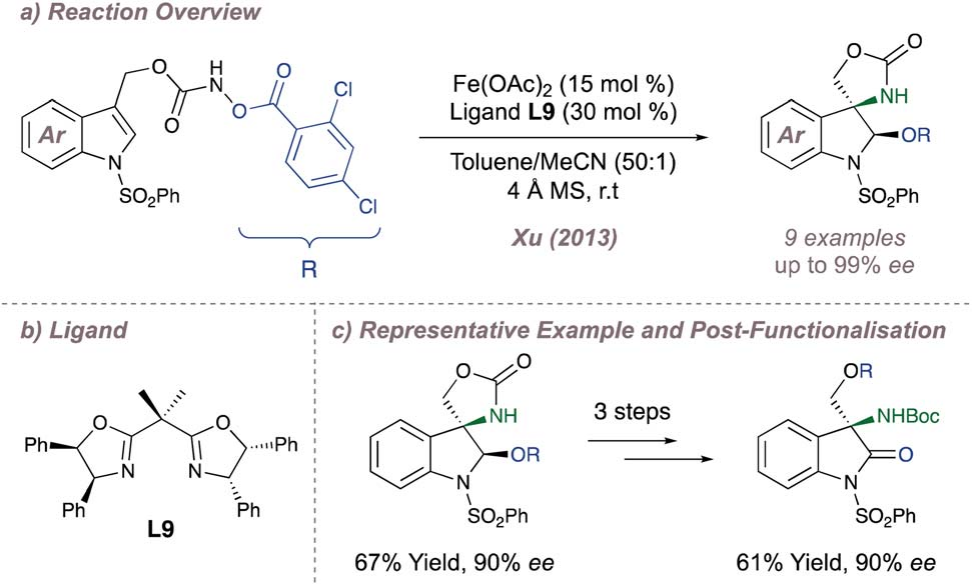
Xu's enantioselective indole aminohydroxylation reaction.

In the realm of iron catalysis, Xu investigated an analogous intramolecular aziridination/ring opening of indoles using hydroxylamines as carbamate nitrene precursors (Fig. 7a).^60^ The advantage of these substrates is that the nitrogen–oxygen bond can be considered an internal oxidant and it removes the need for external hypervalent iodine reagents. Following intramolecular aziridination onto the indole, the strained intermediate is attacked by the nucleophilic alcohol released in the first step leading to formal aminohydroxylation products with good *ee* and *dr*. Extensive optimisation of reaction conditions (particularly with respect to the degree of substitution of the BOX ligand L9) was critical for good reaction outcomes (Fig. 7b) and the products could be further functionalised to enantioenriched amino indolines and amino oxindoles (Fig. 7c). The methodology was later expanded to the asymmetric aminochlorination of styrenes.^61^

Finally, Betley investigated the use of BOX ligands to support nickel iminyl complexes during intramolecular asymmetric azide cyclisations, forming pyrrolidines for which up to 73% *ee* could be obtained.^62^

**Fig. 8:**
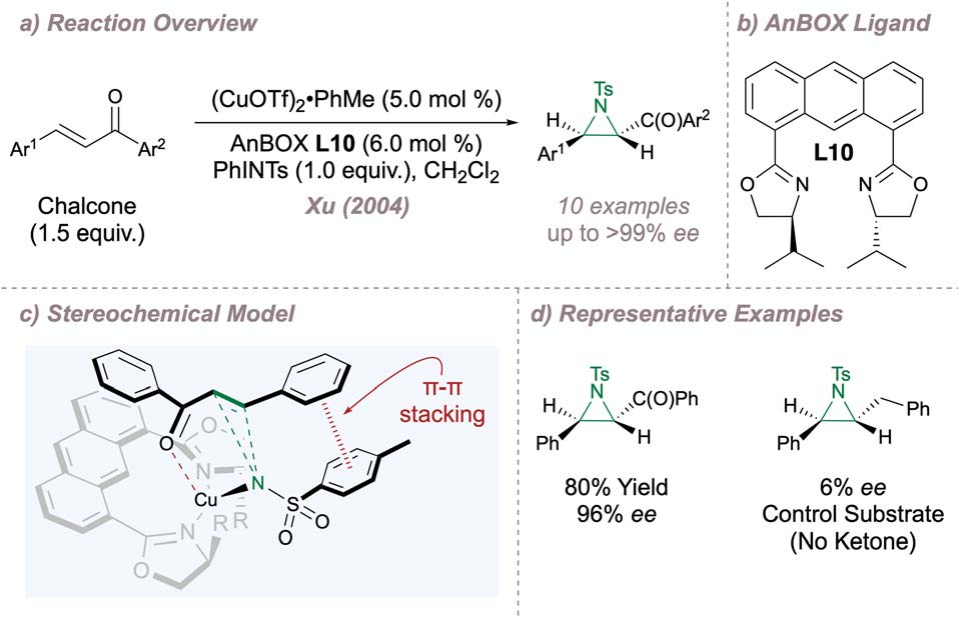
Application of Xu's AnBOX to asymmetric aziridination.

The remainder of this section will examine related designs based on the parent BOX scaffold. In 2004, Xu introduced the 1,8-bisoxazolinylanthracene (AnBOX) family of ligands and chose the copper-catalysed asymmetric aziridination of chalcones to validate the new design (Fig. 8a).^63^ In these ligands, the oxazolines are attached to a rigid anthracyl spacer resulting in a wide bite angle (Fig. 8b). Ligand L10 proved uniquely effective for the transformation, delivering products in up to >99% *ee* although with opposite absolute configuration compared to Evans' system (Fig. 2), despite the fact that in both cases the oxazolines were derived from l-amino alcohols (Fig. 8d). For the Cu(i)-AnBOX system, the metal is positioned above the plane of the spacer (due to rotation of the oxazoline rings) and there is two-point substrate recognition through (i) ketone coordination to copper and (ii) a π–π stack between the aryl group of the copper nitrenoid and the substrate phenyl group attached to the alkene (Fig. 8c).^63,64^ When one of the catalyst–substrate interaction points was removed (for example the ketone carbonyl group) the *ee* fell drastically, lending support to the requirement for two-point binding. Further tuning of ligand bite angle was investigated by replacing the anthracyl with a *trans*-cyclohexane spacer leading to an improved substrate scope for aziridination.^65^

**Fig. 9:**
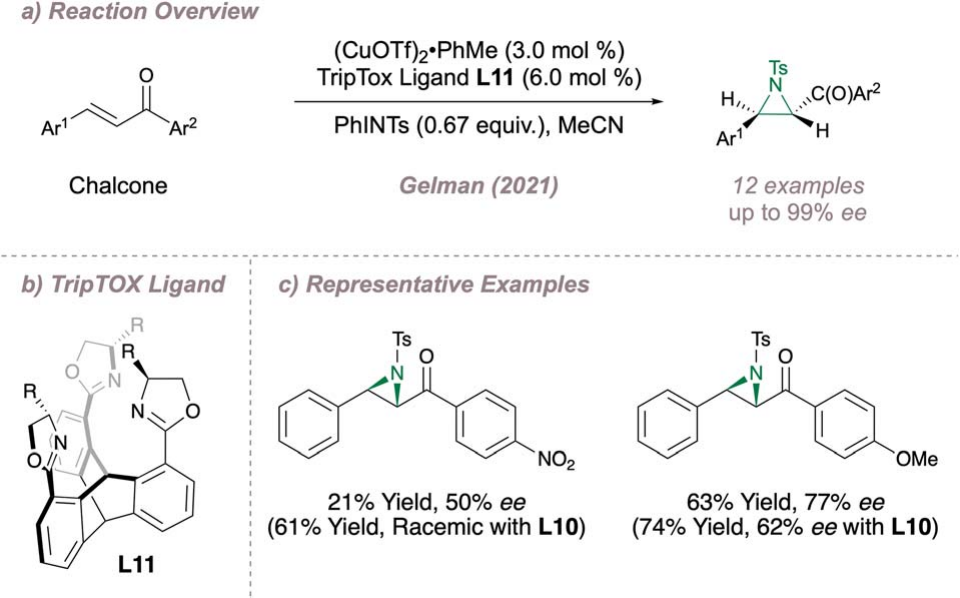
Application of the TripTOX ligand to asymmetric aziridination.

More recently, Gelman reported the design and synthesis of a *C*_3_-symmetric tris(oxazoline) (TOX) ligand L11, also choosing the copper-catalysed intermolecular aziridination of chalcones as the proof-of-concept reaction (Fig. 9a).^66^ In this unique design, three coordinating oxazoline groups are arranged around a rigid triptycene core (TripTOX) (Fig. 9b). Pleasingly, there was complementarity in substrate scopes for the AnBOX and TripTOX catalysed-reactions, with the latter performing particularly well for the aziridination of substrates containing potentially-coordinating functionality such as nitro or methoxy groups (Fig. 9c). The authors rationalised this result by suggesting that the tridentate coordination at copper provided a more discriminating chiral environment with the added advantage of “steric protection” around the metal to prevent unwanted binding by external functional groups.

**Fig. 10:**
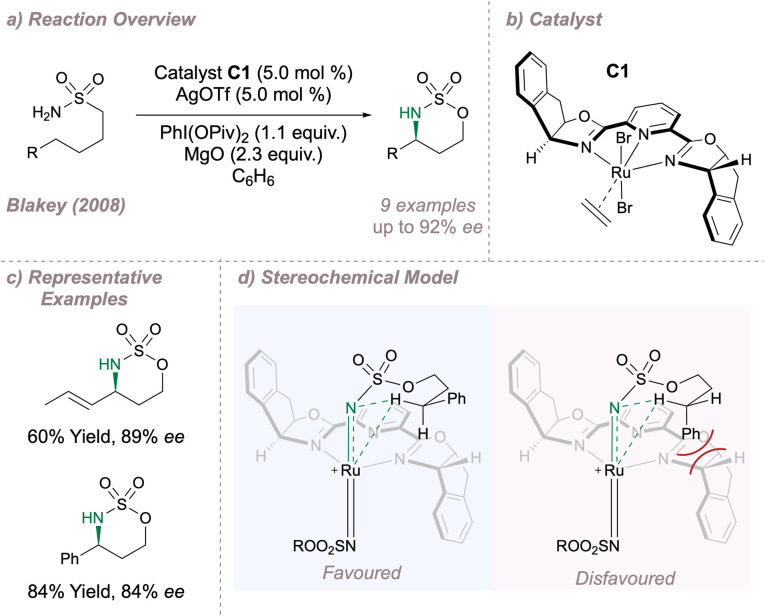
Application of a Ru-(PyBOX) complex for asymmetric intramolecular C(sp^3^)–H amination.

In 2008 Blakey reported one of the first examples in which a chiral ruthenium-pyridine bis(oxazoline) (Ru-(PyBOX)) catalyst C1 was used for asymmetric intramolecular C(sp^3^)–H amination of sulfamates (Fig. 10a and b).^67^ Using Du Bois' optimised amination conditions^23^ along with a halide abstractor to free a metal coordination site and enable nitrenoid formation, intramolecular cyclisation onto activated benzylic or allylic positions was achieved with good enantioselectivity (Fig. 10c). Extension to unactivated positions was not possible since removal of the activating group led to very poor yields. The authors proposed that a cationic bisimido ruthenium complex was the active aminating agent, operating as a triplet nitrene and the stereochemical outcome was rationalised as a result of the minimisation of steric clash between the activating group and the ligand (Fig. 10d).

**Fig. 11:**
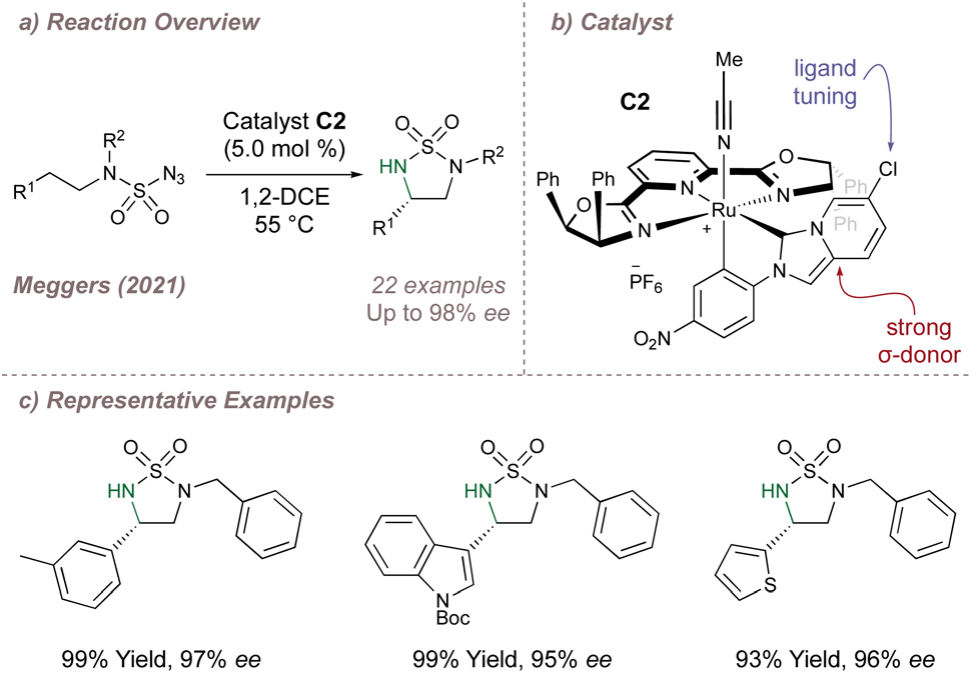
Application of a cyclometallated Ru–PYBOX complex to asymmetric intramolecular C(sp^3^)–H amination.

An important consideration when using PyBOX ligands is that the ligand can withdraw electron density from the metal through π-backbonding. In cases of Lewis acid catalysis this can be advantageous but in other instances it can be detrimental to reactivity. As a solution in the second scenario, Meggers proposed using another ligand to replace the electron density lost to the PyBOX and designed new Ru–PyBOX complexes bearing a strong σ-donating cyclometallated N-Heterocyclic Carbene (NHC) ligand (Fig. 11a and b).^68^ These new complexes were first tested in an enantioselective ‘oxazole to azirine’ ring contraction and a further round of ligand tuning through variation of the substituent at the 3-position of the imidazo[1,5-*a*] pyridine allowed for the application of catalyst C2 to a highly enantioselective intramolecular asymmetric cyclisation of sulfonyl and sulfamoyl azides (Fig. 11c).^69^ Meggers has made other important contributions to the development of asymmetric nitrene transfer reactions and these are discussed in further detail in Section 2.4.

**Fig. 12:**
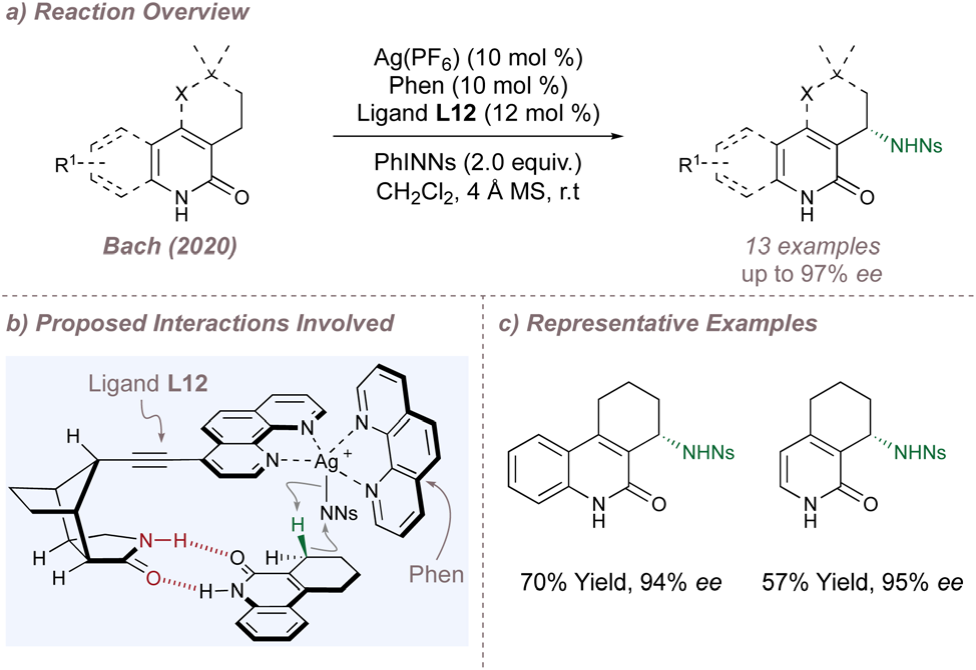
Enantioselective C(sp^3^)–H amination catalysed by a heteroleptic silver complex and directed by hydrogen bonding interactions.

So far, all of the BOX ligands and their derivatives discussed have relied on unfavourable steric repulsion between the substrate and ligand sidearms to disfavour the formation of the minor enantiomer. Bach has explored a different approach in which attractive hydrogen-bonding interactions operating between a related 1,10-phenanthroline ligand L12 and substrate were used to promote the formation of the major enantiomer in a silver-catalysed C(sp^3^)–H amination (Fig. 12a and b).^70^ Incorporation of a chiral lactam recognition unit at the ligand C-4 position enabled two-point hydrogen bonding with 2-quinolone and 2-pyridone substrates, selectively orienting one of the prochiral C(sp^3^)–H bonds towards the heteroleptic silver nitrenoid complex and resulting in high selectivities for nitrenoid insertion (Fig. 12c). This templating approach has been successfully applied by the same laboratory to many other asymmetric processes, including intermolecular nitrene transfer using Rh(ii,ii) catalysis (see Section 2.5).^71^

### 2.2 Chiral diimine and salen ligands

**Fig. 13:**
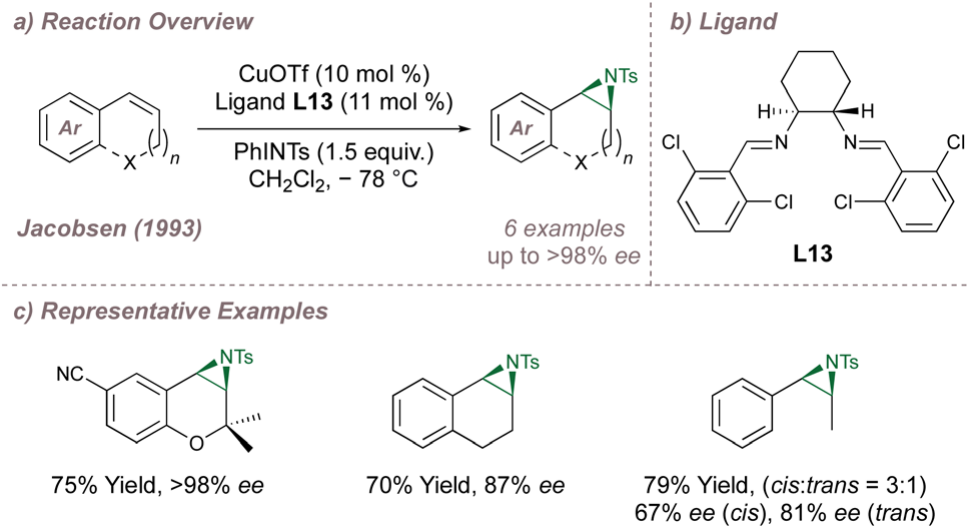
Jacobsen's copper-catalysed asymmetric aziridination of unactivated olefins using a chiral diimine ligand.

At the beginning of the 1990s, the introduction of chiral metallosalen [M(Salen)] complexes revolutionised the field of asymmetric epoxidation by allowing for efficient and undirected oxygen-transfer to simple olefins.^72–75^ It was Burrows who first questioned whether these same complexes would be as effective for the analogous aziridinations.^76^ In the first instance, this was not the case and high-performing [M(Salen)] epoxidation catalysts led only to racemic aziridines making it clear that optimisation would not be as straightforward as simply switching between oxygen and nitrogen sources.^76^ The Jacobsen and Katsuki laboratories were the leaders in the development of the epoxidations^72,77^ and the competition between these groups intensified in the pursuit of an asymmetric aziridination.^78^ In 1993, both groups published protocols for asymmetric aziridination and Jacobsen's initial disclosure in this area is summarised in Fig. 13.^79,80^

Jacobsen started by examining Cu(Salen)-type complexes and, like Burrows, noted poor outcomes. Hypothesising that open coordination sites on copper were essential for high selectivity, a series of bidentate diimines were tested (in place of the tetradentate ligands used previously), with variations in ring substitution and on the diamine backbone. Several ligands were evaluated and it was found that both components affected reaction outcomes. More flexible backbones led to poorer results, potentially due to de-rigidification of the complexes, and aromatic substitution affected catalyst lifetime and selectivity. With optimal ligand L13 (Fig. 13a and b), excellent selectivities could be obtained for the same alkene classes that had proven so amenable to the epoxidation (Fig. 13c). Likewise, the alkenes problematic in epoxidation also performed poorly in aziridination. Mechanistic experiments^81^ suggested that nitrogen transfer is stepwise and occurs through radical intermediates (given that acyclic *cis*- olefins led to a mixture of *cis*- and *trans*- aziridines). Furthermore, there was evidence that the reaction followed a redox mechanism and that ligand accelerated catalysis^82–85^ was operative.

**Fig. 14:**
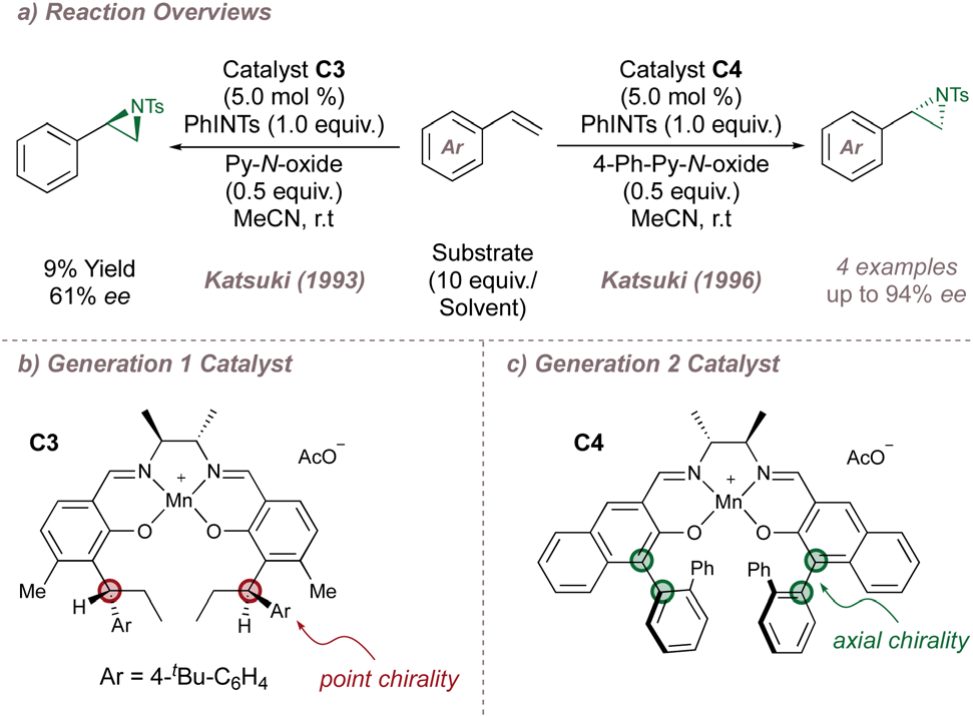
Katsuki's first- and second-generation salen ligands for asymmetric aziridination. Py-*N*-oxide = pyridine-*N*-oxide.

The Katsuki group undertook, over the following two decades, a methodical programme of catalyst refinement which has resulted in the current state-of-the-art salen-based protocols and the course of this progress is summarised here. Katsuki's first report on undirected asymmetric aziridination used first-generation Mn(iii)(Salen) complexes and again compared unfavourably with the asymmetric epoxidation. A few encouraging results were obtained for styrene (up to 61% *ee*) using catalyst C3 which bears a chiral diamine backbone and point chirality on the aromatic rings of the ligand (Fig. 14a and b).^80^ An important advance was made in 1996, inspired by a breakthrough in catalyst design for the asymmetric epoxidation: replacement of the point chirality elements in catalysts such as C3 (Fig. 14b) with planar chirality as for C4 (Fig. 14c) had led to much higher epoxidation selectivities and faster reaction rates.^86,87^ When applied to aziridination, this insight immediately improved the yield and *ee* for the functionalisation of styrenes with a ligand-acceleration effect noted also (Fig. 14a).^88^

Alongside catalyst design, Katsuki also investigated methods to make nitrene transfer more environmentally-friendly. The use of iminoiodinanes or hypervalent iodine reagents is problematic as these exhibit low atom-economy and generate unwanted by-products. In 2003 Katsuki found that in the presence of Ru(Salen) catalysts, sulfonyl azides could act as effective nitrene precursors.^89^ Not only is nitrogen gas the only by-product, but UV irradiation was not required for nitrene formation. Good performance was observed under these conditions although the chemoselectivity between aziridination and C(sp^3^)–H amination was unpredictable. At around the same time, Che was also investigating aziridination using Ru(Salen) complexes and reported a promising enantioselective aza-Rubottom reaction to afford enantioenriched α-amino ketones.^90^

**Fig. 15:**
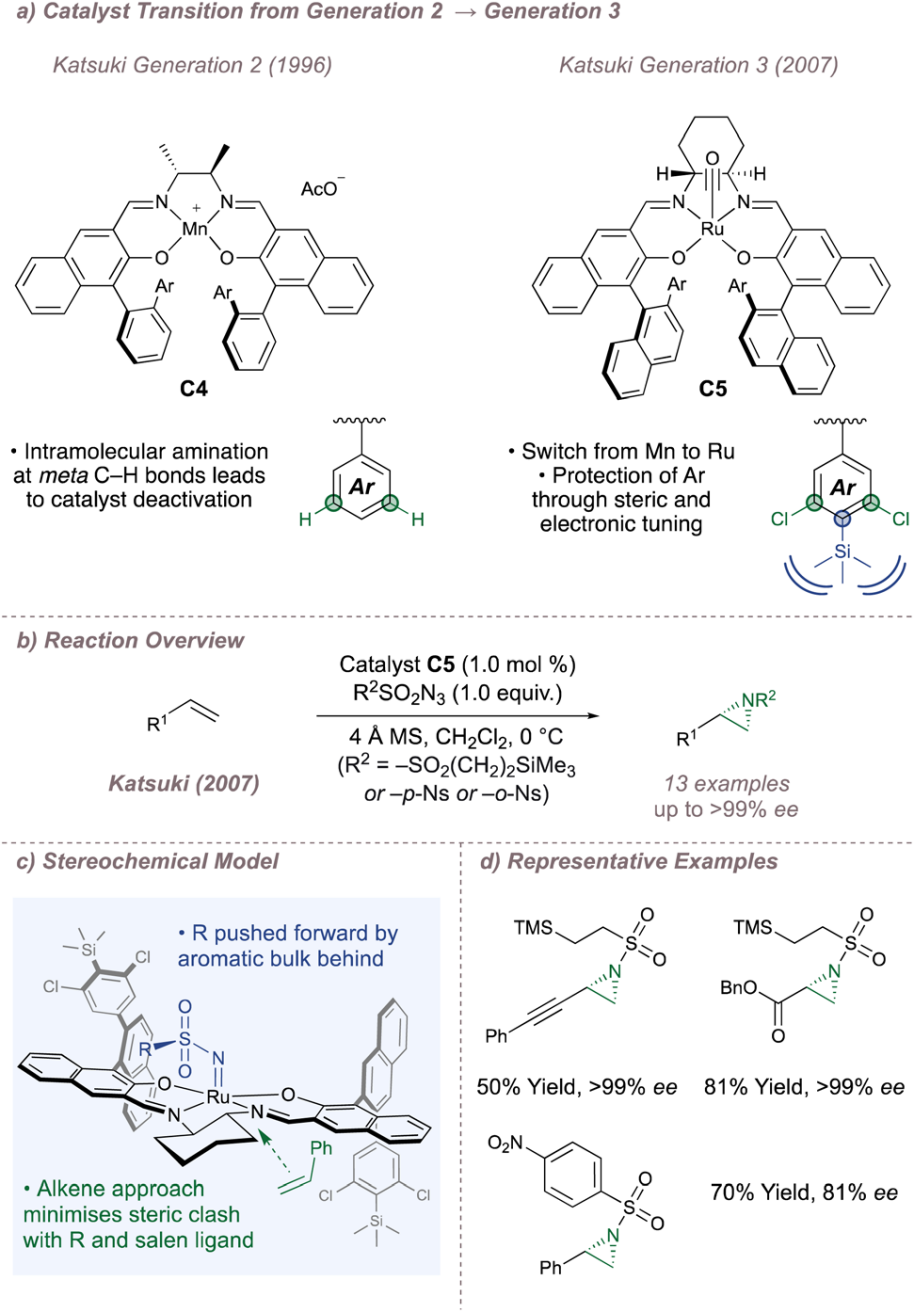
Progression from second- to third-generation M(Salen) catalysts.

From 2004 onwards, Katsuki combined insights into catalyst design with the “greener” nitrene precursors and, in doing so, significantly upgraded the methodology. During previous studies on sulfimidation, it was noted that the salen catalysts aminated themselves at the *meta* carbon of the phenyl substituents on the 3- or 3′- position to give complexes which were inactive to nitrene transfer.^91^ In order to counter this, approaches based on either steric blocking (by introducing bulky substituents at the *para* position) or replacement of the *meta* C–H bonds with inert halogens were investigated (Fig. 15a).^92^ Both were successful and these more robust third-generation catalysts boasted higher turnover numbers (up to 878) and broadened scope (especially towards unactivated terminal alkenes such as 1-octene). In particular, when steric blocking and halogenation were applied together (C5), turnover numbers and substrate scopes continued to improve with excellent *ee*s now observed across a range of activated and unactivated alkenes (Fig. 15b). Thanks to these modifications, azide precursors (such as NsN_3_ and SESN_3_) which are less reactive but easier to deprotect^93,94^ could now be used, compromising neither yield nor *ee*.^95^ In addition, the olefin could be introduced as a limiting reagent instead of as a co-solvent.

With the third-generation Ru(Salen) complexes, a stereochemical model (which was guided by X-ray crystal structures obtained for similar complexes) was introduced to explain the facial selectivity of olefin functionalisation (Fig. 15c).^96^ As with the epoxidation, Katsuki suggested a side-on approach of the alkene to the metal centre but for the aziridination, matters are complicated by the steric bulk (RO_2_SN−) of the sulfonyl group which is absent in the analogous metal-oxo species. The 2′′-aryl substituent at the back left of the ligand as drawn is close to the metal nitrenoid forcing the steric bulk on nitrogen forwards. In turn, the larger substituent on the incoming olefin is placed away from it which leads to the stereochemistry shown. By 2012, further catalyst fine-tuning resulted in highly efficient systems capable of functionalizing a broad range of unactivated alkenes with excellent metrics (Fig. 15d).^97,98^

**Fig. 16:**
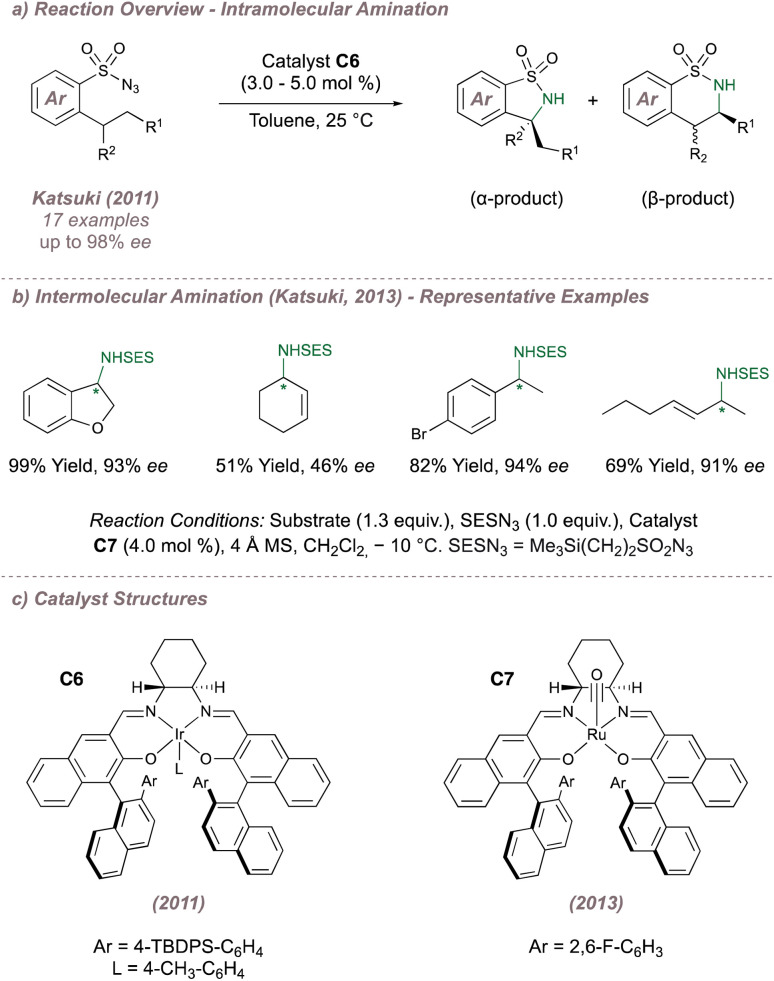
Application of M(Salen) catalysts to C(sp^3^)–H amination.

During studies on asymmetric aziridination, Katsuki had occasionally noted the formation of the allylic C(sp^3^)–H amination products,^89^ particularly in cases where electron deficient M(Salen) complexes were employed.^99^ More recent investigations from this group focussed on improving the enantioselectivities for C(sp^3^)–H amination by first examining the intramolecular reaction at benzylic positions using sulfonyl azides as precursors to sultams (Fig. 16a). Third-generation Ir(iii)(Salen) complex C6 (Fig. 16c) mediated the cyclisation with excellent yields and enantioselectivities, although in some cases unpredictable product mixtures arising from functionalisation at the α- and β- positions were observed. The more challenging intermolecular amination at benzylic and allylic positions was then studied using Ru(CO)(Salen) C7 (Fig. 16b and c).^100,101^ Excellent enantio- and regioselectivities were obtained for the amination of 23 feedstock alkenes and the highlight of the work was the ability of the catalyst to effectively discriminate between groups such as ethyl and *n*-propyl, which are sterically almost identical. The authors took this opportunity to probe whether the intermolecular amination with the proposed metal nitrenoids proceeded *via* a concerted or stepwise mechanism. Experiments with substrates bearing either built-in radical clocks, or adjacent olefins capable of undergoing radical isomerisation were carried out. In both instances no cyclopropane ring opening or olefin isomerisation was observed and as a result a concerted mechanism was cautiously proposed. It should be noted however, that a stepwise mechanism could not be entirely ruled out given that radical rebound might be extremely rapid.

**Fig. 17:**
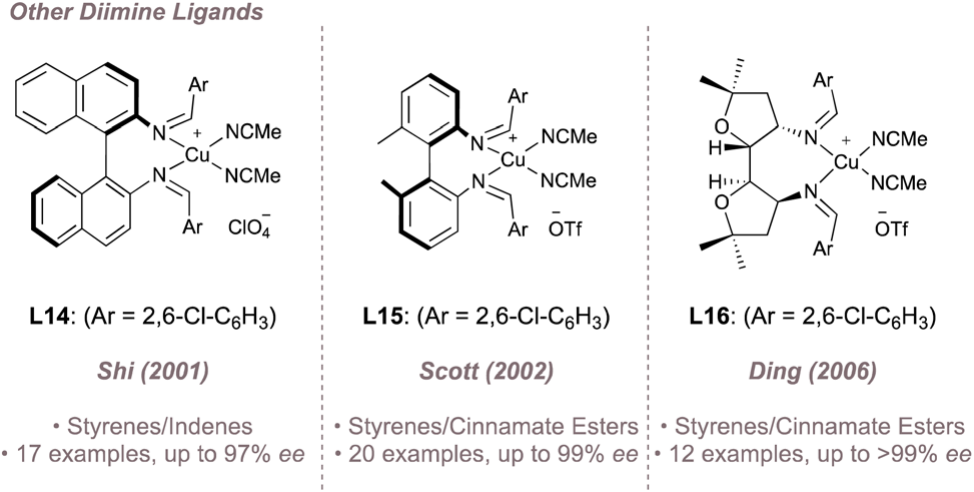
Other copper-diimine complexes used for asymmetric aziridination.

While salen ligands are undoubtedly the lead class of diimines for asymmetric nitrene transfer, other designs (typically used as ligands for copper) have also been reported for asymmetric aziridination albeit displaying limited reaction scope. Examples of such ligands (L14–L16) complexed to copper are shown in Fig. 17.^102–104^

### 2.3 (Cp*)M(iii) complexes

(Cp*)M(iii) complexes are capable of catalysing a diverse range of C–H functionalisation reactions, including asymmetric C(sp^3^)–H amination.^105–110^ Aminations using these catalysts can proceed through either inner sphere or outer sphere mechanisms and examples of both are discussed below.^111^ In order to maintain focus, only transformations that form the chiral centre α-to the amine will be considered.

Rendering inner-sphere (Cp*)M(iii)-catalysed processes enantioselective is inherently difficult because at the crucial C–H activation step the metal is coordinatively saturated. Therefore, conventional asymmetric strategies such as the ligation of the metal by chiral phosphines cannot be used to induce asymmetry. This idiosyncratic problem has been partially solved thanks to generations of elaborate chiral cyclopentadienyl ligand designs based on “steric wall” approaches or even catalyst incorporation within the chiral environment of an enzyme active site.^112^ This has allowed for effective enantioinduction in certain (Cp*)M(iii)-catalysed reactions with the drawback that the synthesis of such catalysts is often non-trivial. Yoshino and Matsunaga have investigated an alternative approach in which an achiral, cationic Cp*M(iii) complex is associated with an external anionic chiral source (such as a chiral sulfonate or carboxylate anion) with the latter responsible for the enantioinduction.^113,114^

**Fig. 18:**
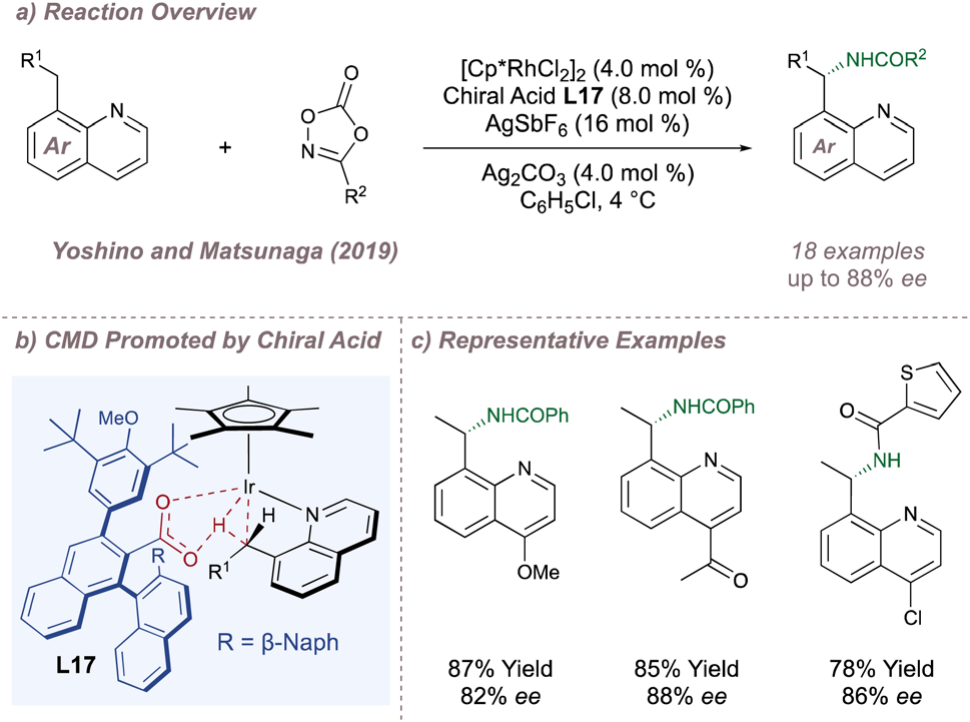
Enantioselective amination of 8-alkyl quinoline through enantiodetermining CMD.

In the realm of asymmetric C(sp^3^)–H amination this concept was demonstrated in the functionalisation of 8-alkyl quinoline substrates wherein insertion of the (Cp*)Rh(iii) at the benzylic C–H position is enantiodetermining (Fig. 18a).^115^ Given that the C–H activation proceeds *via* carboxylate-assisted concerted metalation-deprotonation (CMD) it was hypothesised that a chiral carboxylic acid could be used to render this step enantioselective while maintaining only monodentate coordination to the metal (Fig. 18b). BINOL-derived carboxylic acid L17 was optimal and a range of quinoline substitutions were tolerated. The C–N coupling with the nitrenoid is downstream of the enantiodetermining CMD, so substitution on the nitrogen source had little impact on *ee*. This in turn allowed for the transfer of nitrogen from several different dioxazolone precursors (Fig. 18c). Ellman later reported a similar reaction using one of Cramer's second-generation Cp* ligands^112^ and with substrates bearing an oxime directing group in place of the basic quinoline nitrogen.^116^

**Fig. 19:**
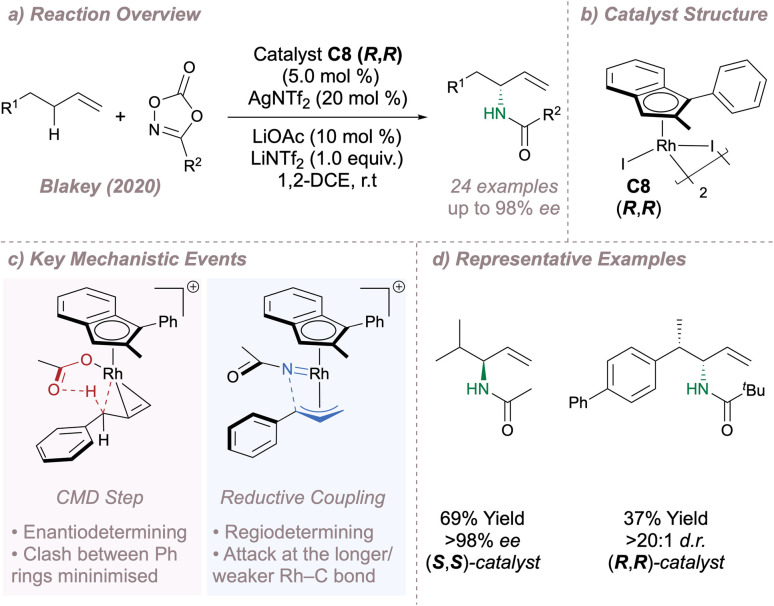
Blakey's asymmetric allylic amination of alkene feedstocks.

In 2020 Blakey reported a planar chiral Rh(iii)-indenyl catalyst C8 for regio- and enantioselective allylic C–H amination (Fig. 19a and b).^117^ In this case no directing groups on the substrate are required, enabling a highly selective reaction with feedstock olefins. The use of a chiral indenyl ligand operating through steric and electronic effects was key. The indenyl can bind to the metal through η^5^- or η^3^- coordination modes with the latter exerting a greater *trans*-influence. Regio- and enantioselectivity are therefore determined as a result of an unusual “electronic asymmetry” ligand effect since one end of a bound π-allyl fragment has a longer Rh–C bond than the other. C–H activation is the enantiodetermining step and is also influenced by steric effects which serve to minimise the repulsion between the substrate and ligand (Fig. 19c, left). The fact that 2-pentene and 1,3-diphenylpropene both afford products with high enantioselectivity is telling – at first glance reactions using these substrates would be expected to proceed through symmetrical π-allyl intermediates. However, the high product *ee*s derived from these starting materials lend support to an effective transmission of chiral information from the ligand to the substrates *via* the subtle “electronic asymmetry” effect as opposed to a more conventional strategy which relies on the minimisation of steric clash alone. Following CMD, dissociation of the acetate leaves a free coordination site at Rh. The dioxazolone binds at this site, releasing CO_2_ to form the Rh(v) nitrene which undergoes reductive C–N coupling. The coupling is under kinetic control, occurring preferentially at the Rh–C bond that has been weakened by the indenyl *trans*-influence. Further, steric clashing between the imide and the indenyl is also minimised, reinforcing the desired regioselectivity (Fig. 19c, right). Excellent results were obtained across different nitrogen sources and alkenes to afford useful building blocks with potential for further transformations (Fig. 19d).

**Fig. 20:**
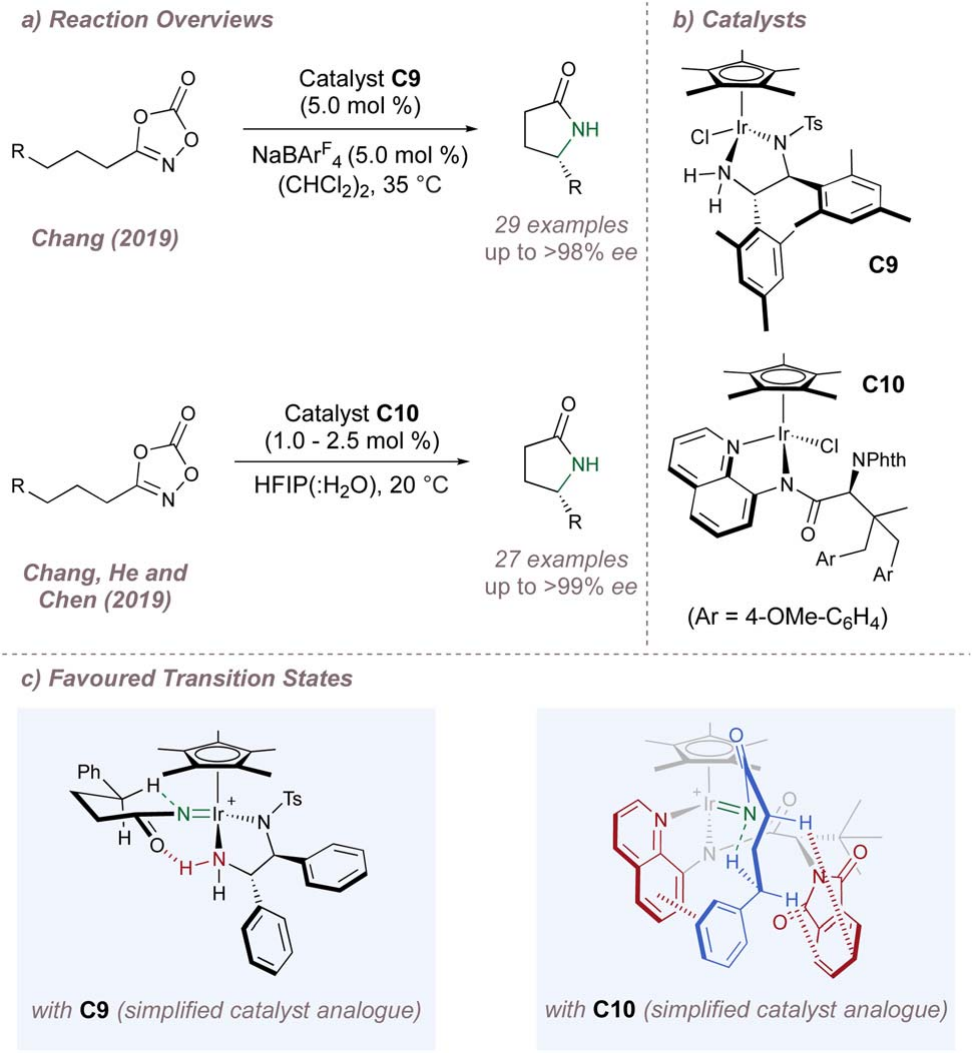
Asymmetric intramolecular amination of dioxazolones showcasing different chiral ligand designs.

Moving now to the outer-sphere processes, the Chang group has made significant contributions to the development of (Cp*)M-catalysed reactions and has pioneered outer-sphere aminations, invoking attractive non-covalent interactions between substrates and ligands to influence all aspects of selectivity.^118^ In 2019, this group reported the intramolecular amination of dioxazolones to afford chiral lactams using (Cp*)Ir(iii) complex C9 (Fig. 20a and b, upper).^119^ (An identical transformation was published simultaneously by the Yu group using Ru(ii) catalysis also invoking enantiocontrol through hydrogen bonding).^120^ Excellent enantioselectivities were observed for functionalisation at both activated and deactivated positions and the competing Curtius rearrangement was effectively supressed.^121^ DFT calculations demonstrated that in the lowest–energy transition state, a hydrogen-bond between one of the diamine protons (acidified through *N*-coordination to iridium) and the carbonyl oxygen not only facilitates dioxazolone decarboxylation but also stabilises the C–H insertion which proceeds with the large chain substituent in the pseudoequatorial position (Fig. 20c, left). Given the importance of the postulated hydrogen bond for both enantioinduction and reactivity the authors investigated this interaction further. Since isolation of the putative Ir-nitrenoid would have been very challenging due to its instability, the neutral and far-more stable Ir-amido complex was studied as a model system instead. For this compound, evidence of the key hydrogen bond was obtained in solution (through a significant downfield shift of the relevant proton observed by ^1^H NMR) and also in the solid state through an analysis of interatomic distances and bond angles between the suggested hydrogen bond donor and acceptor. To further bolster the hypothesis, it was found that disruption of the ideal hydrogen bond through either mono- or di-methylation of the amine led to progressively poorer *ee*. Taken together, these three pieces of evidence convincingly support the stereochemical model derived from computations. Whereas in this case, a metal nitrenoid surrogate was used as a model system, very recent and exciting work from the same group has demonstrated the possibility of isolating and studying acyl nitrenoids themselves using crystalline matrix isolation techniques.^122^ The further development of similar methods will undoubtedly yield critical insights into the mechanism and potentially the mode of enantioinduction for metal nitrenoid transfers in general.

Almost immediately after, Chang, He and Chen reported another cyclisation reaction but this time using the entirely different catalyst C10 (Fig. 20a and b, lower).^123^ Again, DFT studies suggested that a network of attractive non-covalent interactions was responsible for the high selectivities observed (Fig. 20c, right). The Cp*, aminoquinoline and phthalimide groups of the ligands shroud the iridium centre within a chiral hydrophobic pocket which readily accommodates the substrates given the polar reaction medium. Following nitrene formation, intramolecular insertion proceeds through a chair transition state stabilised through both π–π stacking and C–H/π interactions between the substrate and catalyst. The pocket surrounding the metal is extremely effective at accommodating the transition state for intramolecular cyclisation across a range of saturated substrates. In order to probe the key proposed catalyst–substrate C–H/π interaction identified by the computations, catalysts bearing alternative nitrogen protecting groups in place of the phthalimide were tested. In particular, the use of benzamide or carbamate protecting groups (which possess more flexibility and a less extended aromatic system compared to the phthalimide) led to a reversal in the sense of enantioinduction (potentially as as result of acting through a steric blocking mode alone) and to much poorer *ee* values.

**Fig. 21:**
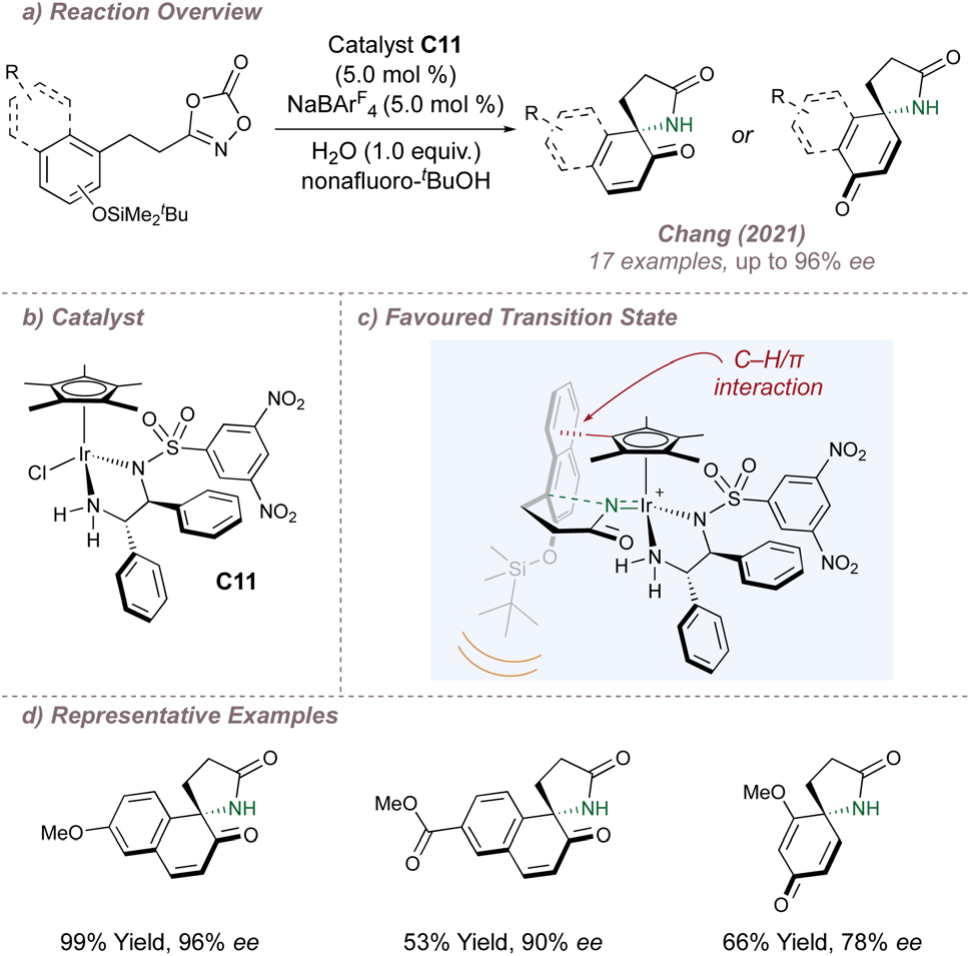
Chang's asymmetric spirolactamisation of dioxazolones.

Chang has continued to explore these intramolecular aminations, extending the scope to formal allylic C(sp^3^)–H amination^124^ and alkene aminofunctionalisation.^125^ Of particular note is the recent report of intramolecular nitrenoid cyclisation onto protected naphthols to generate spirolactams (Fig. 21a, b and d).^126^ Once again, non-covalent interactions, this time operating between catalyst C11 and the substrate, are responsible for the high selectivity. C11 bears the familiar diamine ligand but DFT now suggested that a different set of attractive non-covalent interactions were operative compared to above (Fig. 21c). In the favoured transition state, the naphthol ring is oriented downwards to avoid steric clash between the bulky – OTBDMS and the ligand. In this conformation, attractive C–H/π interactions can also operate between the Cp* and the extended aromatic system. In the absence of the bulky silicon “auxiliary” very poor enantioinduction was observed, highlighting the combined contribution of steric and electronic effects in determining selectivity. The Chang group continues to make important advances in these outer-sphere amination reactions, most recently through a multidimensional catalyst screening approach of their vast metal-precursor and ligand libraries.^127^

### 2.4 Chiral-at-metal catalysts

**Fig. 22:**
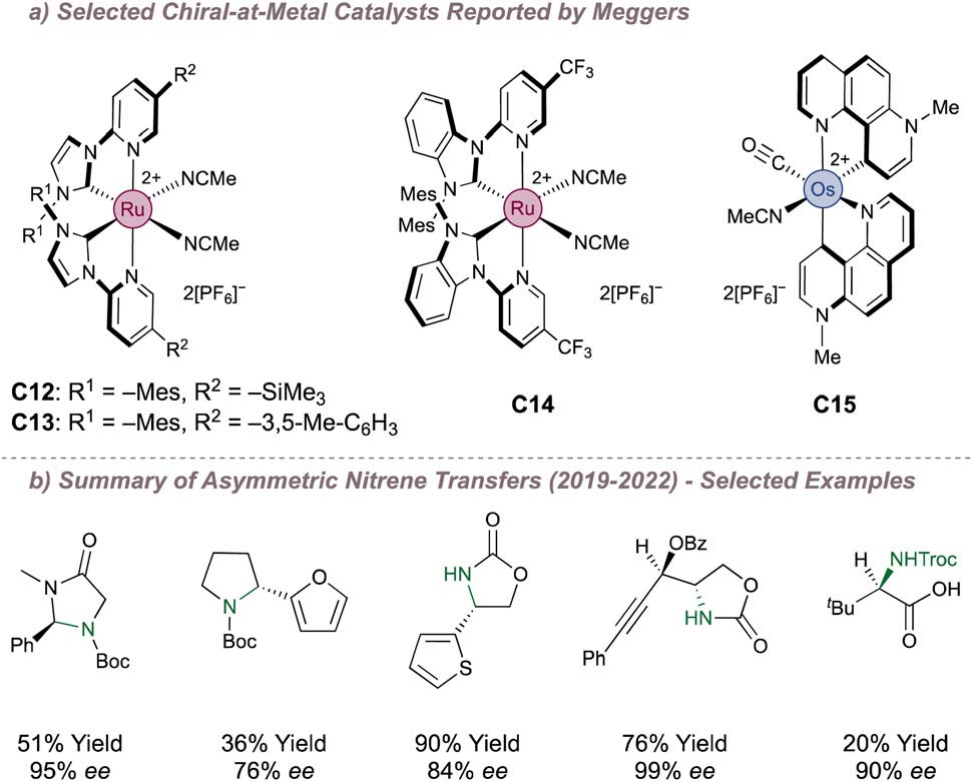
Catalysts reported by Meggers for asymmetric nitrene transfer.

In the examples examined so far, asymmetric induction for nitrene transfer has often arisen through placing the chirality in the metal coordination sphere in the form of bound chiral ligands. A lesser-used strategy but one with great potential, is to instead make the metal itself a stereogenic centre. In an octahedral complex, the metal atom defines an origin about which ligands (not necessarily chiral) can be assembled. The complex as a whole can be chiral depending on whether a left-handed (Λ-configured) or right-handed (Δ-configured) helix arises from the ligand arrangement.^128^ The group of Meggers in particular has done much to introduce this type of complex into mainstream catalysis by first reporting efficient routes to synthesise the enantiopure complexes^129,130^ and then applying these to various asymmetric transformations.^131–134^ Of particular interest are the more recent reports in which these catalysts have been used for asymmetric nitrene transfer. Fig. 22a shows examples of relevant complexes to this end and highlights the structural features which can be used to tune their performance. Over the last three years, Meggers has methodically investigated these for (predominantly) intramolecular nitrene transfer using azide or *N*-benzyloxycarbamate precursors (Fig. 22b). This has resulted in efficient protocols, predominantly for the formation of various enantioenriched saturated heterocycles.^135–140^ Two examples will now be examined in further detail; Meggers' first pioneering report describing the cyclisation of 2-azidoacetamides as well as a recent publication which reports a powerful platform for asymmetric amino acid synthesis.

**Fig. 23:**
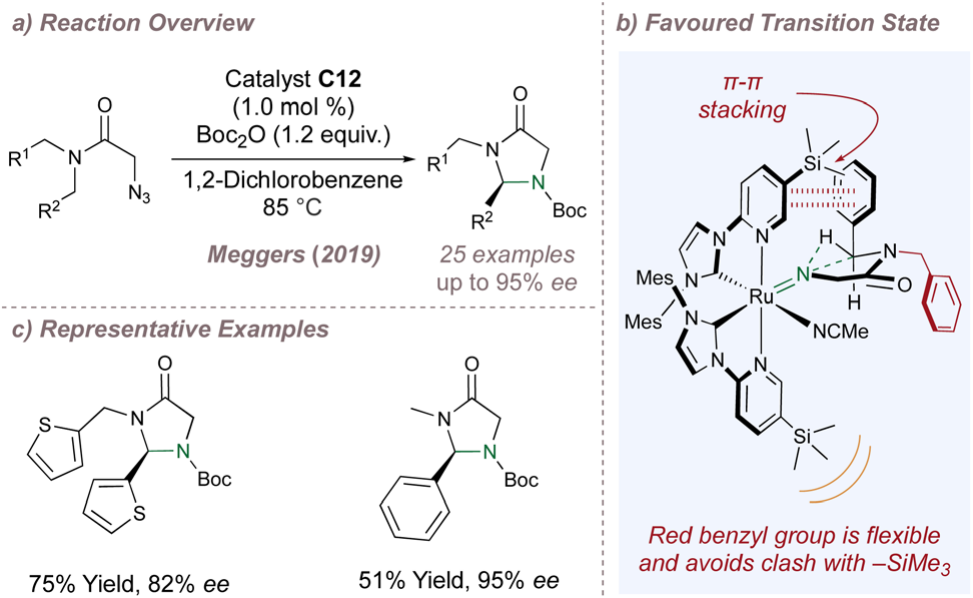
Meggers' first asymmetric amination using chiral-at-metal catalysts.

Meggers chose the cyclisation of 2-azidoacetamides to chiral imidazolidine-4-ones as a validation reaction for asymmetric intramolecular nitrene transfer catalysed by chiral-at-metal complexes (Fig. 23a).^135^ For this particular reaction, detailed investigation of the metal and bulky substituent at the 3-position of the pyridine ring led the authors to catalyst C12 (Fig. 22a). Other chiral-at-metal catalysts were completely ineffective and an *N*-Boc protection step following cyclisation prevented catalyst poisoning by the free amine products.

Mechanistically, dissociation of the labile acetonitrile ligands from the catalyst enables two-point substrate binding through the carbonyl and –N_3_ groups. Following loss of nitrogen, a ruthenium–imido complex forms which, after dissociation of the carbonyl, allows the free singlet nitrene to insert into the chain. Support for the crucial two-point binding mode was obtained through reaction of an archetypal azide substrate with an equimolar amount of racemic catalyst at room temperature instead of at the elevated reaction temperatures. At the lower temperatures the C–H insertion did not occur and quantitative formation of a chelated imine complex was observed instead. This complex was characterised by X-ray crystallography and was thought to arise from a precedented sequence of azide coordination and tautomerisation through a 1,2-H shift. The majority of the substrates were tested in desymmetrisation mode in which R^1^ = R^2^ = Ar (Fig. 23c). In addition to activating the C–H bond to insertion, the aromatic group on the substrate is also proposed to engage the pyridine ring of the ligand in a π–π stack at the favoured transition state. The more flexible –CH_2_R^1^ group attached to the amide nitrogen atom is oriented downwards and has enough conformational freedom to avoid clash with the bulky –SiMe_3_ on the underside of the complex (Fig. 23b). Overall, the enantioselectivities were excellent and this reaction provided a solid foundation for the many other asymmetric cyclisations subsequently reported by the Meggers group.

**Fig. 24:**
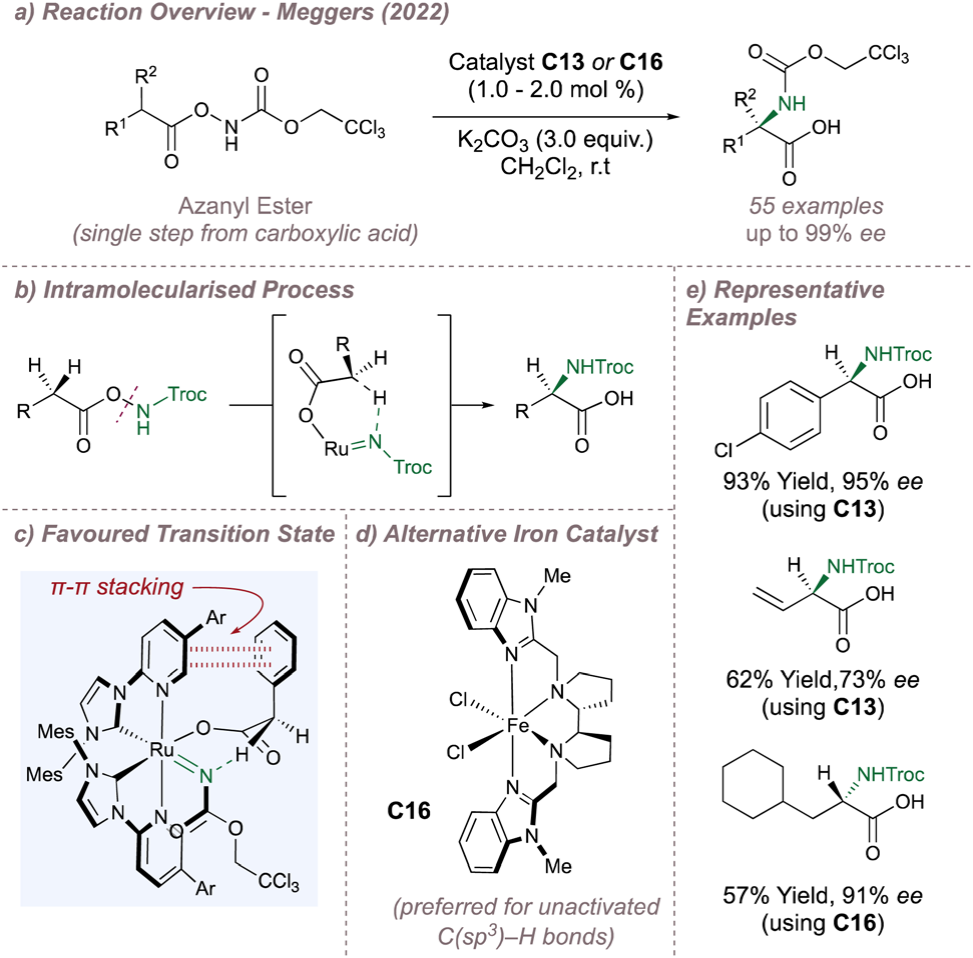
Meggers' asymmetric synthesis of enantioenriched α-amino acids.

In 2022, Meggers moved away from intramolecular cyclisations and reported an exciting advance for the synthesis of enantioenriched α-amino acids (Fig. 24a).^140^ The following reaction lies somewhat in between an inter- and intramolecular process and incorporates the more desirable features from each. Intramolecular nitrene transfers benefit from the high selectivities that arise from cyclic transition states but, given that the products themselves are also cyclic, opportunities for further derivatisation can be limited. In contrast, intermolecular amination affords more versatile acyclic protected amines but control over selectivity can be more challenging.

In this example, a Troc-protected azanyl ester (obtained in a single step from the corresponding carboxylic acid) serves as a nitrene source by undergoing N–O bond cleavage in the presence of the catalyst to leave both fragments bound to the central metal atom (Fig. 24a and b). An intramolecular cyclic transition state accommodated within the helical chirality of catalyst C13 then leads to control over the site-selectivity and enantioselectivity of the insertion (Fig. 24c). Using ruthenium catalysis, excellent yields and selectivities were obtained for amination at activated (benzylic, allylic and alkynyl) positions with high diastereoselectivities obtained also when using enantiopure substrates matched with the chiral catalyst configuration (Fig. 24e). However, C13 gave poor activity for nitrene insertion into unactivated C(sp^3^)–H bonds. Fortunately, (*R*,*R*)-FeBIP C16, a catalyst which is not chiral-at-metal but has a similar overall topology to C13 was excellent for this more challenging insertion (Fig. 24d). The two catalysts are complementary since conversely C16 struggles in the functionalisation of the activated C(sp^3^)–H bonds. DFT calculations suggested that *N*-insertion proceeds *via* a triplet nitrene whereby the spin crossover/radical recombination step is faster than radical interconversion. As with the previous example, an attractive π–π stacking interaction between the substrate and the 3-pyridyl group results in high *ee* although when the substrate aromatic group is replaced by an alkyl group, C–H/π interactions may operate instead. In addition to the excellent selectivity and wide substrate scope, the Troc protecting group is easy to remove rendering this a highly practical method. Very recently, Meggers reported a fully-intermolecular variant of this reaction using carboxylic acid substrates and BocNHOMs as the nitrene source. The catalyst of choice was C16 and the substrate scope and enantioselectivities for the enantioenriched Boc-protected α-amino acid products were excellent.^141^

### 2.5 Dirhodium tetracarboxylate and tetracarboxamidate complexes

Rh(ii,ii) tetracarboxylate and tetracarboxamidate paddlewheel dimers are often the catalysts of choice for performing nitrene transfer. Before considering enantioselectivity, a brief overview of their catalysis is warranted. These dimers are excellent Lewis acids and for a long time have been known to decompose diazo compounds to form rhodium carbenes.^142,143^ This reactivity has been studied extensively by Doyle and Davies (amongst many others) who have made seminal contributions to enantioselective and site-selective carbene transfer using these catalysts.^13,144–151^ In particular, the control over carbene insertion using Davies' rhodium paddlewheels is unparalleled and firmly places this method amongst the most versatile C–H functionalisation protocols.

**Fig. 25:**
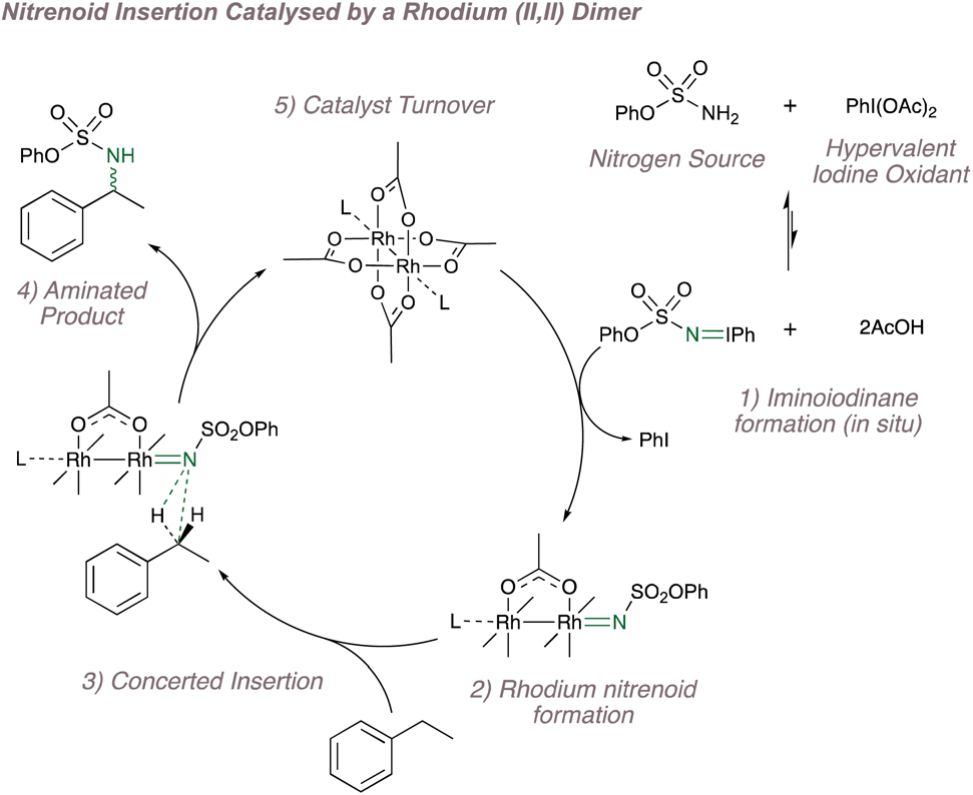
A catalytic cycle for C(sp^3^)–H amination catalysed by a Rh(ii,ii) dimer.

These same dimers can also decompose common nitrene precursors (such as iminoiodinanes, azides and *N*-tosyloxycarbamates) to form the rhodium nitrenoids which show parallel reactivity to the carbenoids as discussed previously (see Fig. 1).^152^ A simplified cycle for Rh(ii,ii)-catalysed nitrene transfer, featuring *in situ* iminoiodinane formation, is shown in Fig. 25.^153^ (Note that the dimer has been drawn in full in subheading 5 but is shown in abbreviated form in the other steps: these representations are equivalent and the abbreviation will be used hereon in for convenience). In addition to *in situ* formation of the nitrene precursor, reactions using these catalysts have the added advantage that they proceed under mild conditions, frequently under an atmosphere of air and are not affected by ambient moisture, all factors that have encouraged their widespread uptake.^153,154^

While there are clear parallels between carbenoid and nitrenoid chemistry, asymmetric nitrene transfer using these dimers has so far fallen short of the standard set by the carbene-transfer protocols since functionalisation can currently only be achieved at electronically activated positions.

**Fig. 26:**
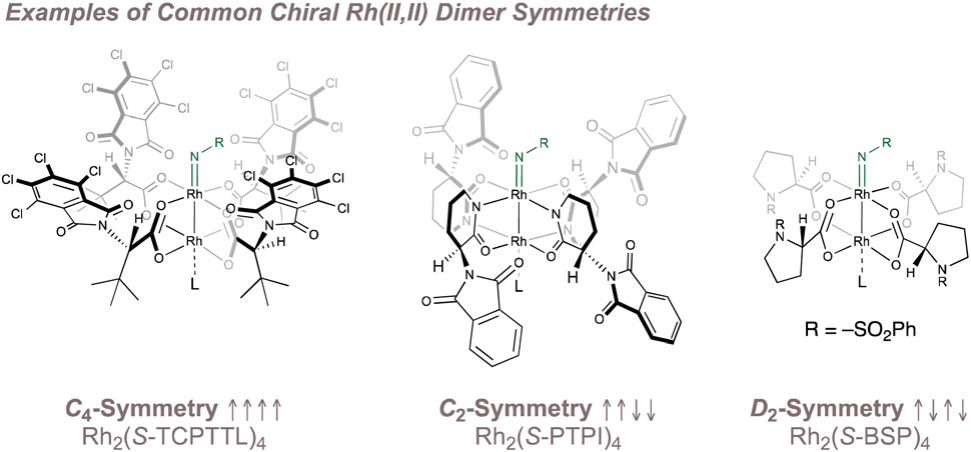
Common Rh(ii,ii) dimer symmetries.

The unique structure of the paddlewheel complexes has led to innovative approaches for inducing asymmetry in their catalysis. Given the fourfold ligand arrangement about the dirhodium core, the most common strategy exploits this pattern to build a higher order of symmetry in the overall complex (typically *C*_4_, *C*_2_ or *D*_2_) from four chiral *C*_1_-symmetric ligands.^145,146,155,156^ Examples of common chiral Rh(ii,ii) dimer symmetries are shown in Fig. 26 with the *C*_4_ chiral crown/bowl “all up” ligand arrangement predominating for asymmetric nitrene transfer reactions. Other highly-creative strategies to render rhodium paddlewheels chiral have been reported^156,157^ but only those that have been applied to enantioselective nitrene transfer are discussed. In addition, methods in which enantiopure nitrogen sources are used in conjunction with chiral rhodium dimers to perform diastereoselective aminations^158^ are not considered as they fall outside the scope of this review.

**Fig. 27:**
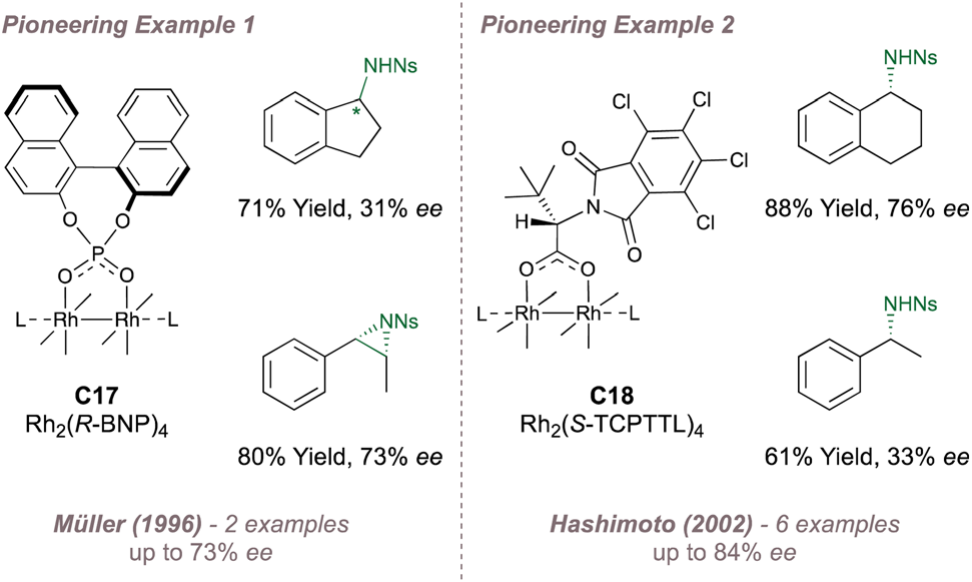
Early application of chiral Rh(ii,ii) dimers to asymmetric nitrene transfer.

Two pioneering examples of asymmetric amination using Rh(ii,ii) catalysis are now presented. The first was reported by Müller in 1996 ^159^ who investigated Pirrung's catalyst^160^C17 for the asymmetric amination of indane and asymmetric aziridination of styrene and *cis*-β-methylstyrene (Fig. 27, left). The results were encouraging although the scope was limited. A later report in which the same intermolecular amination was re-examined alongside intramolecular aziridination led to slightly enhanced selectivities but with ample room for improvement (<60% *ee*).^161^ The second example was reported by Hashimoto in 2002 who introduced catalyst C18 incorporating four (*S*)-TCPTTL carboxylate ligands which are derived from *tert*-leucine and bear electron-withdrawing chlorinated *N*-phthaloyl protecting groups (Fig. 27, right).^162^ This catalyst showed improved selectivity for amination compared with Müller's system but more importantly, most of the other paddlewheel catalysts for enantioselective intermolecular nitrene transfer are derived from this particular design.

**Fig. 28:**
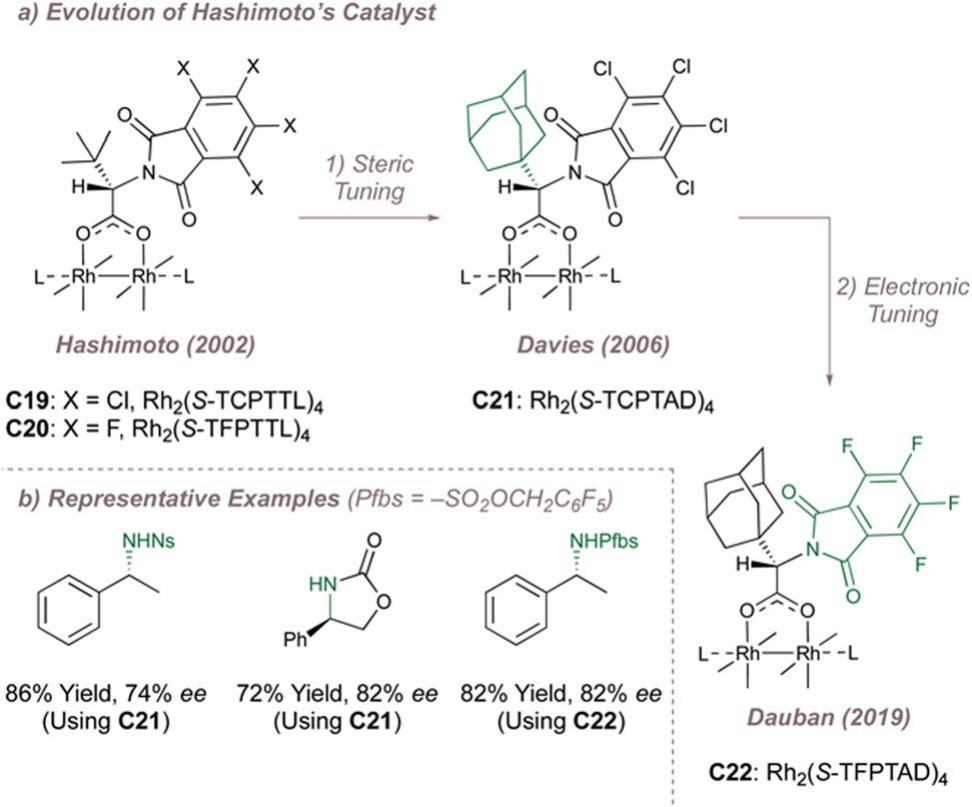
Evolution of Rh_2_(*S*-TCPTTL)_4_ and application to various asymmetric nitrene transfer reactions.

In the first instance Davies replaced the *tert*-butyl groups in Rh_2_(*S*-TCPTTL)_4_ with even larger 1-adamantyls to form Rh_2_(*S*-TCPTAD)_4_C21 (Fig. 28a, centre).^163^ This improved the *ee* for indane amination but even seemingly innocuous changes to this substrate were not particularly well tolerated (Fig. 28b). The same catalyst did however perform slightly better in an intramolecular amination mode. Dauban was next to modify Hashimoto's parent catalyst by replacing the chlorine atoms with more withdrawing fluorines while retaining the bulkier admantyl leading to Rh_2_(*S*-TFPTAD)_4_C22.^164,165^ This catalyst was applied to the very challenging intermolecular benzylic amination of simple ethylbenzene derivatives using a polyfluorinated aromatic nitrogen source (Fig. 28a and b).^164,165^

**Fig. 29:**
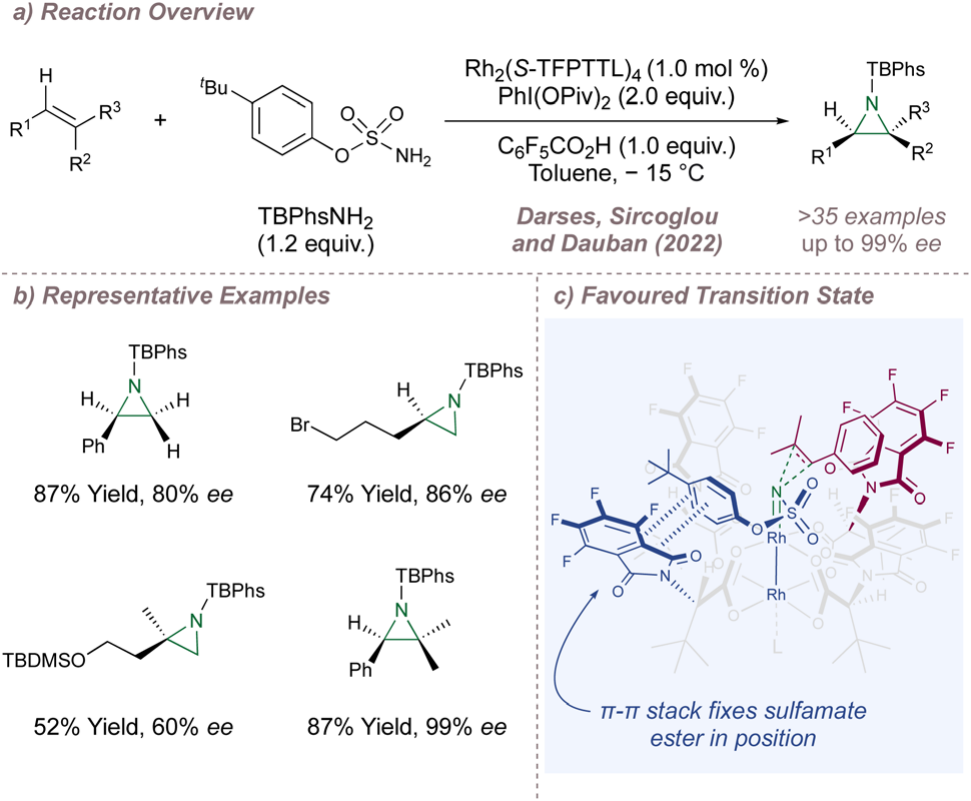
Asymmetric aziridination of terminal alkenes and trisubstituted styrenes catalysed by Rh_2_(*S*-TFPTTL)_4_.

Next, Darses, Sircoglou and Dauban investigated the use of Hashimoto's polyfluorinated Rh_2_(*S*-TFPTTL)_4_C20 for intermolecular aziridination (Fig. 29a).^166^ This reaction is at the very forefront of enantioselective Rh(ii,ii)-catalysed nitrene transfer and broadened the scope of asymmetric aziridination in general to highly-substituted styrenes, an under-represented substrate class in the earlier protocols. The enantioselectivities obtained for the typical terminal styrene substrates were good, but the incorporation of a β,β-dialkyl group onto the terminal carbon atom improved the selectivity significantly. Remarkably, terminal alkyl olefins were also excellent substrates (Fig. 29b). A detailed computational study elucidated the mode of enantioinduction (Fig. 29c). A π–π stacking interaction between one of the phthaloyl groups and the aromatic ring of the sulfamate ester locks the conformation of the latter (Fig. 29c, blue) within the chiral bowl. The remaining space around the nitrenoid is then very well-suited to accommodating *tri*-substituted styrenes.

**Fig. 30:**
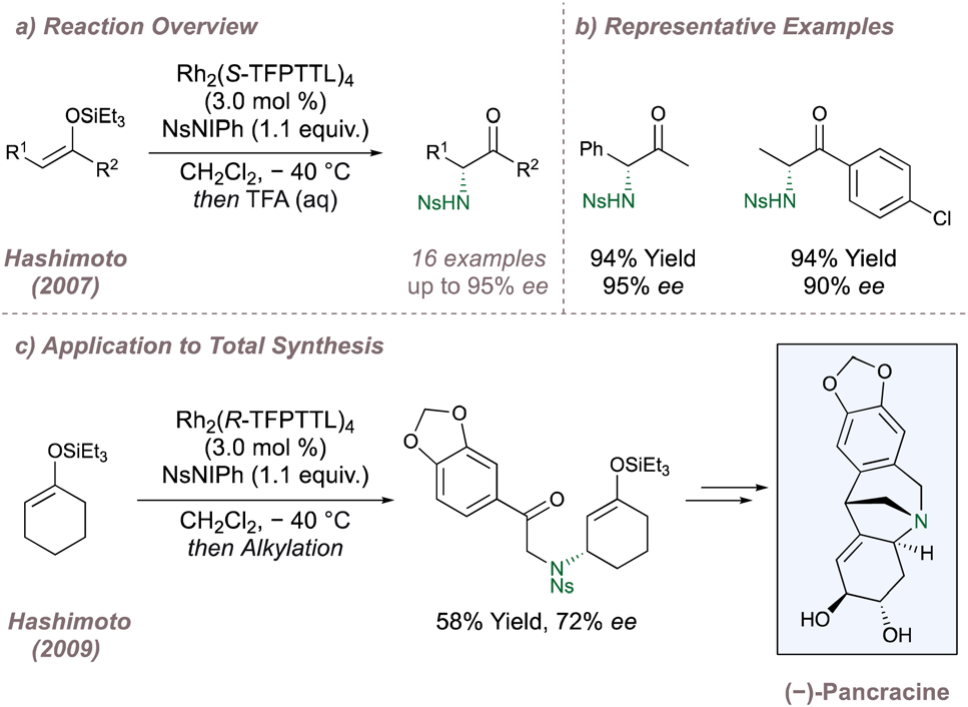
Enantioselective aza-Rubottom oxidations and allylic aminations using chiral Rh(ii,ii) paddlewheel dimers.

The asymmetric intermolecular protocols discussed in this section so far have largely focussed on nitrogen-transfer to positions adjacent to aromatic rings. However, as the field has developed, other transformations have been reported. One such example is the asymmetric aziridination of *Z*-silyl enol ethers which is immediately followed by silyl deprotection/aziridine ring opening to afford valuable α-amino carbonyls (Fig. 30a). These asymmetric “aza-Rubottom” reactions have been studied by Hashimoto, affording products with uniformly high yields and selectivities (Fig. 30b).^167,168^ Upon accommodation of the silyl enol ether within a ring, exclusive allylic amination in up to 72% *ee* was observed instead (Fig. 30c).^169^ This switch in chemoselectivity was ascribed to locking of the alkene in an *E*-geometry with the *E*-isomers unreactive in the previous “aza-Rubottom” reaction. The reaction was used as the first step in the total synthesis of (−)-pancracine with this first chiral centre determining the rest of the relative stereochemistry for the route (Fig. 30c). Corey has also studied the asymmetric intermolecular amination of cyclic silyl enol ethers during investigations into the synthesis of ketamine analogues but observed the formation of the expected α-amino ketone with excellent selectivity.^170^ Another adventurous transformation was recently reported by Yoshida and involved an enantioselective aminative desymmetrisation of 1,3-disubstituted adamantanes using Rh_2_(*S*-TCPTTL)_4_.^171^ Up to 85% *ee* was observed and the resulting amino acids built around an adamantane core could be of considerable biological interest.

**Fig. 31:**
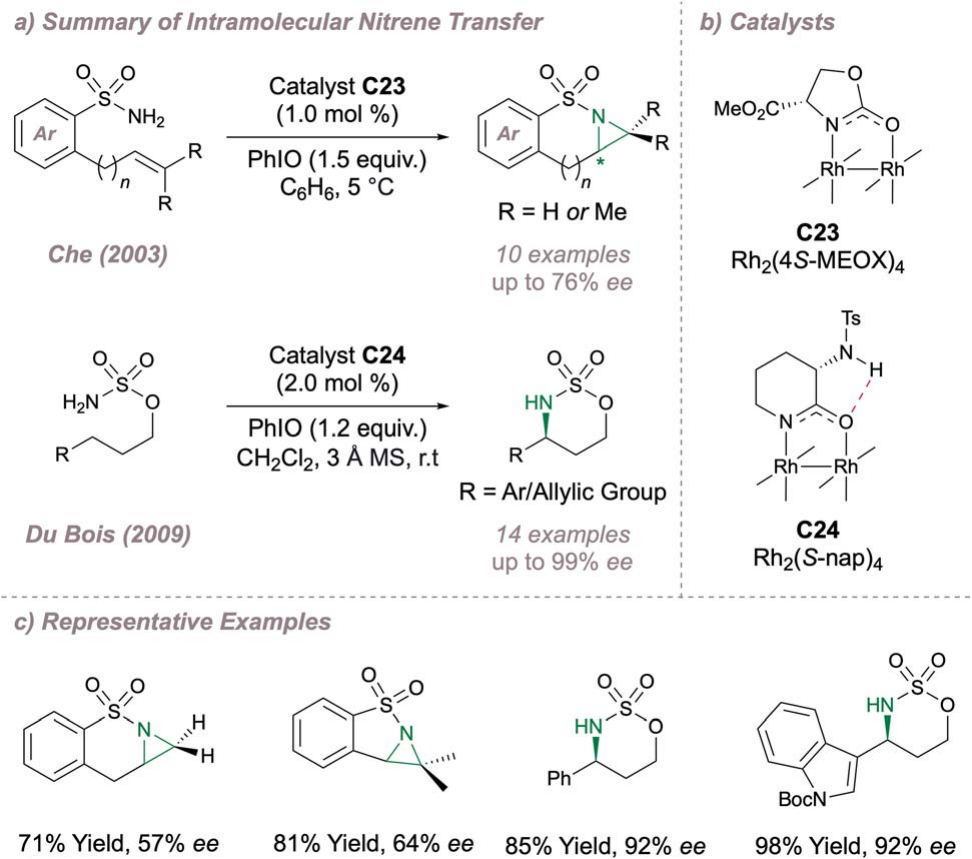
Enantioselective intramolecular nitrene transfer using asymmetric Rh(ii,ii) catalysis.

Intramolecular cyclisation reactions have also been investigated using these dimers. In 2003, Che disclosed an intramolecular aziridination, predominantly of sulfonamides, using Doyle's Rh_2_(4*S*-MEOX)_4_ catalyst C23 originally developed for carbene transfer.^145,172^ Using this method, fused bicyclic aziridines could be obtained with moderate selectivity (Fig. 31a–c). A more selective intramolecular C–H amination protocol was later reported by Du Bois (Fig. 31a–c).^173^ For this transformation the (4*S*-MEOX) ligand was poor, potentially due to crowding around the axial site during the key C(sp^3^)–H insertion. Instead, an alternative amide-derived paddlewheel, Rh_2_(*S*-nap)_4_C24 (which is more resistant to undesired ligand dissociation compared to the carboxylate-ligated dimer) displayed improved performance, delivering products in up to 99% *ee*. Proposed intramolecular hydrogen-bonding in the ligand (Fig. 31b) was thought to be critical in raising the oxidation potential for the undesired conversion of the active Rh^2+^/Rh^2+^ dimer to the catalytically-inactive Rh^2+^/Rh^3+^ under the oxidising reaction conditions. In fact when this hydrogen bond was removed through methyl capping, the *E*_ox_^(ii/ii)→(ii,iii)^ decreased from 330 mV to 242 mV with an accompanying drastic drop in reaction performance highlighting the importance of this subtle catalyst design element. Furthermore, this particular chiral catalyst displays unusual chemoselectivity in that it favours allylic amination over intramolecular aziridination with certain substrates. In both asymmetric intramolecular reactions, more electron-donating amide ligands were favoured in place of carboxylates leading in turn to a less electrophilic (and potentially more selective) rhodium nitrenoid. Along the lines of varying the electronic properties of these catalysts, Yoshino and Matsunaga investigated Du Bois' cyclisation using *C*_2_- and *C*_4_- symmetric diruthenium carboxamidate paddlewheels, obtaining excellent selectivities also.^174^ The overall cationic Ru(ii,iii) dimer is more electron deficient compared to the neutral Rh(ii,ii) making it more resistant to oxidation as confirmed through cyclic voltammetry studies. More recently, these dinuclear ruthenium catalysts have been successfully used for the synthesis of α-amino ketones through an “aza-Rubottom” reaction.^175^ Together with Hashimoto's prior observations as to the importance of polyhalogenated aromatic rings on the catalysts, these examples highlight that both steric and electronic factors can greatly impact selectivity outcomes when using bimetallic paddlewheel dimers.

**Fig. 32:**
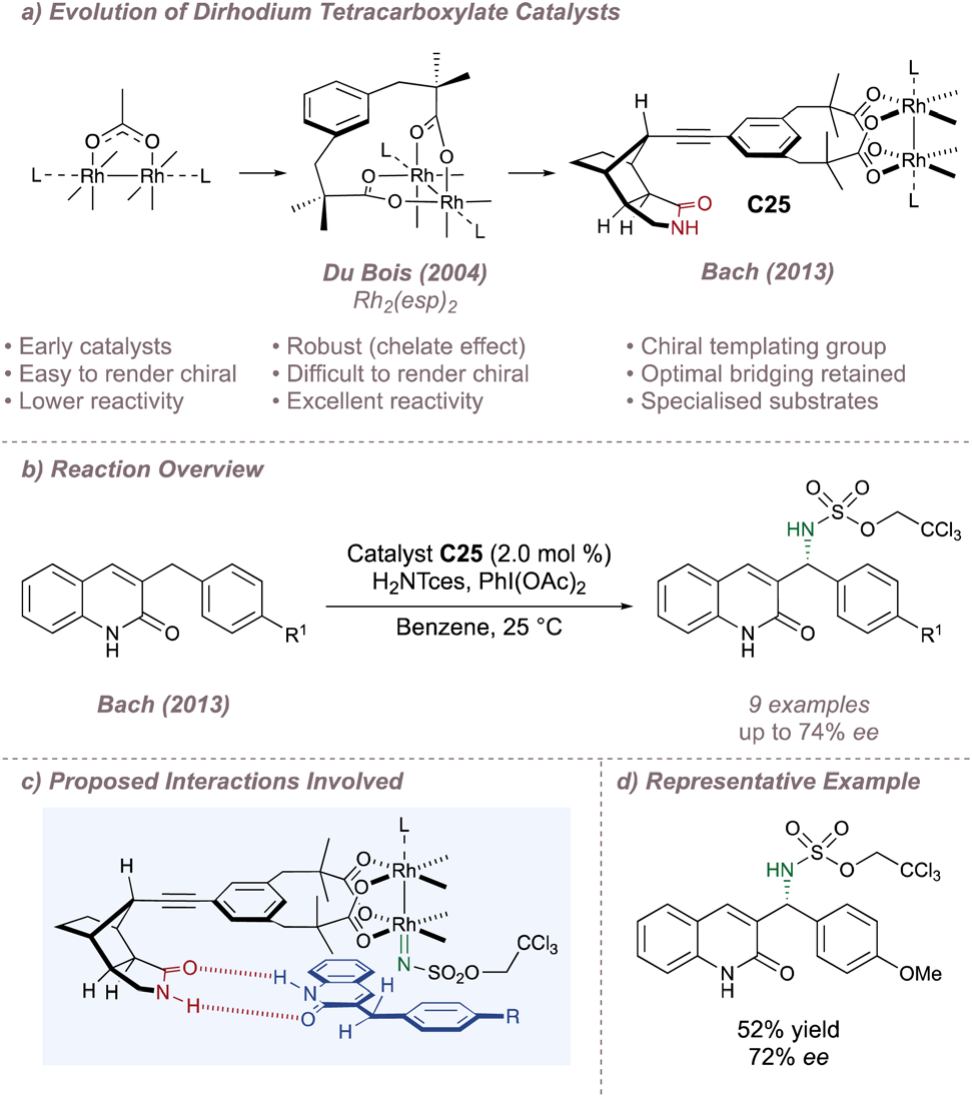
Advent of Rh_2_(esp)_2_ and Bach's development of an effective chiral variant.

The Rhodium dimers discussed so far have consisted of four separate ligands assembled around the bimetallic core. However, under typical amination conditions, there is a tendency for one or more of these ligands to dissociate in a process that initiates eventual catalyst decomposition.^22–24^ This can be a serious problem for effective enantioinduction, particularly if the catalyst degradation products are also capable of catalysing the reaction but with very low selectivity compared to the original dimer.^173,176^ Inspired by work from Taber^177^ and Davies^178,179^ in the field of enantioselective carbenoid insertion chemistry, Du Bois linked together pairs of these carboxylates in a strapping ligand designed to perfectly bridge the distance between the metal ligation sites.^22^ The chelate effect renders the resulting achiral Rh_2_(esp)_2_ complex remarkably stable to ligand exchange. Mechanistic studies have been used to rationalise the superiority of this catalyst for intermolecular amination and it has become the flagship dimer for nitrene transfer using limiting substrate (Fig. 32a).^23,24^

Despite its high activity, the Achilles heel of Rh_2_(esp)_2_ and of strapped Rh(ii,ii) tetracarboxylates in general is that they are inherently difficult to render chiral. Elegant design exceptions include Davies' strapped dirhodium tetraprolinates,^178,179^ May's binaphthyl-based dimer^180^ and Ball's dicarboxylate strapped peptide ligands^181–184^ but these have not been applied to asymmetric nitrene transfer. Compared to the more typical design strategies, the ascent to a higher symmetry is not feasible for strapped dirhodium tetracarboxylates and attempts to incorporate blocking elements can be challenging. On the one hand the steric bulk will need to be placed close to the metal for effective asymmetric induction which runs the risk of destabilising the dimer and lowering its catalytic activity. On the other, if the steric bulk is to be incorporated without straining the dimer it will likely end up far from the metal centre and will be ineffective for inducing asymmetry. Bach recognised these challenges, and turned to an approach based on attractive non-covalent interactions to develop a chiral version of Rh_2_(esp)_2_ (Fig. 32a).^185^

Bach appended a chiral lactam hydrogen bonding recognition unit to the *meta* position of each bridging aromatic ring, linked through a directional alkyne spacer to form *C*_2_-symmetric catalyst C25 (Fig. 32a and b).^185^ An X-ray crystal structure revealed that the U-turn of the rigid lactam pointed the recognition portion of the dimer back towards the axial rhodium site, well-positioned to orient a suitable substrate for enantioselective amination. A similar strategy was discussed previously for an asymmetric silver-catalysed amination reported by the same group (Section 2.1). 3-Benzylquinolones were thought to engage the catalyst through dual hydrogen-bonding thus positioning the pro-(*R*) C(sp^3^)–H bond for amination with up to 74% *ee* (Fig. 32b–d).^185^ Higher enantioselectivities (up to 95% *ee*) were obtained using the catalyst in aziridination mode on analogous alkenes.^186^ Although the substrates are necessarily specialised, these two examples are highly significant as they demonstrated that it was possible to develop chiral variants of Rh_2_(esp)_2_ without compromising the parent catalyst's exceptional reactivity.

Our group has explored an alternative strategy to render the Rh_2_(esp)_2_ scaffold chiral.^187^ In our approach, the achiral paddlewheel dimer is modified through the addition of anionic benzyl sulfonate groups at the *meta* position of both bridging aromatic rings. Each sulfonate then associates with a chiral cation derived from a cinchona alkaloid through ion-pairing. Prior examination of the X-ray crystal structure data for Rh_2_(esp)_2_ suggested that by placing the pendant sulfonates at these positions, the associated ion-paired chiral cations would be capable of forming a discriminating pocket around the axial sites.^22^ In addition to defining the relative position of the chiral cation, the sulfonate was also envisaged to act as a recognition site by engaging appropriate incoming substrates through hydrogen bonding. In a broader sense, the direct association of chiral cations with transition metals is a relatively underexplored strategy since it is rare for the relevant catalytic cycles to contain anionic metal intermediates.^188–190^

**Fig. 33:**
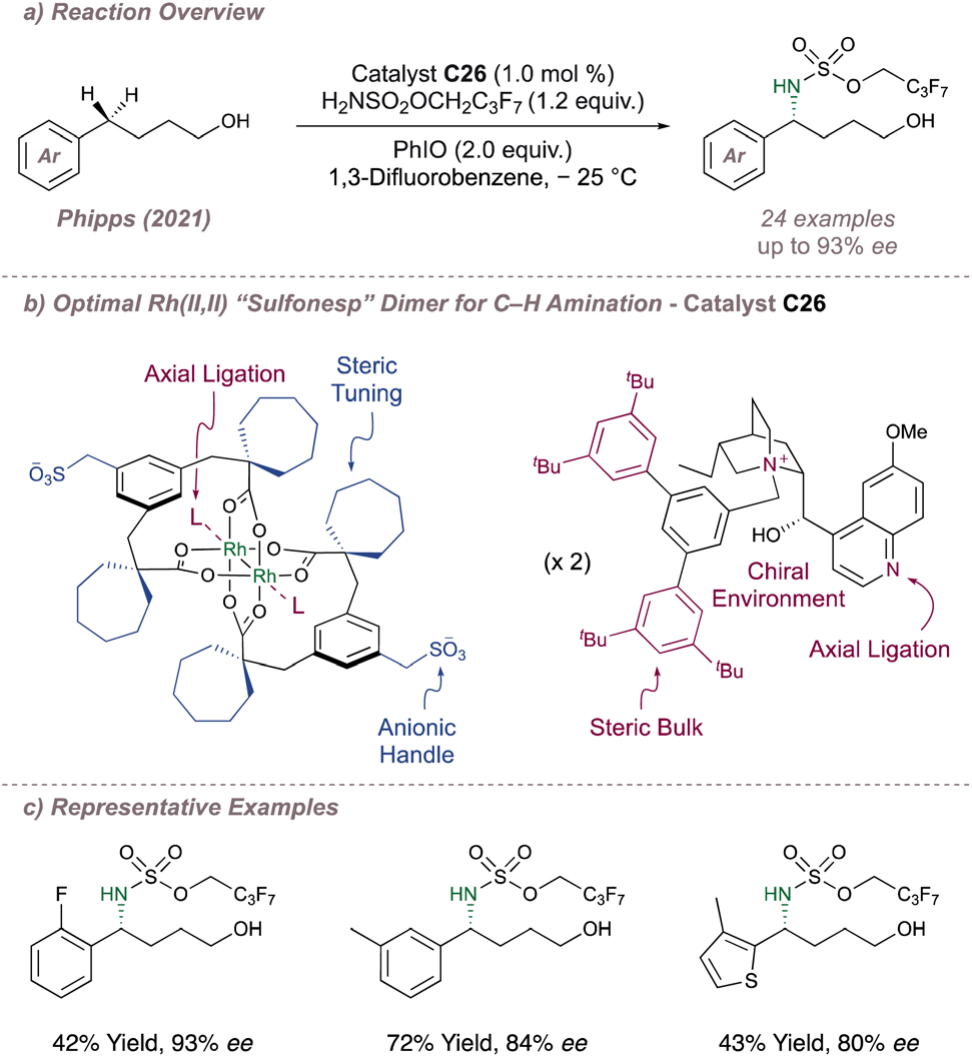
Application of chiral rhodium “Sulfonesp” dimers to the asymmetric benzylic amination of 4-arylbutan-1-ols.

We began by synthesising a library of chiral dirhodium “Sulfonesp” catalysts where the anionic scaffold and chiral cation portions were prepared separately and united in the final step by means of a simple salt exchange. This afforded flexibility in catalyst design by enabling a “mix and match” of scaffolds and cations with the ultimate goal of exerting full catalyst control over selectivity. With a library in hand we next evaluated the catalysts for the enantioselective intermolecular benzylic amination of 4-arylbutan-1-ols (Fig. 33a and b). These substrates were chosen since the terminal hydroxyl group of the alcohol was hypothesised to be key in achieving transition state organisation through hydrogen bonding with the anionic sulfonate. Reaction optimisation identified ion-paired catalyst C26 as the best catalyst for the amination of this substrate class and delivered enantioenriched protected 1,4-amino alcohol products with moderate to good enantioselectivities (Fig. 33c). In addition to their role in inducing enantioselectivity, UV-visible spectroscopy studies suggested that the chiral cations were ligating axially to the dimer which may be partly responsible for the significantly improved product yields obtained with the chiral catalysts compared to when using Rh_2_(esp)_2_.

**Fig. 34:**
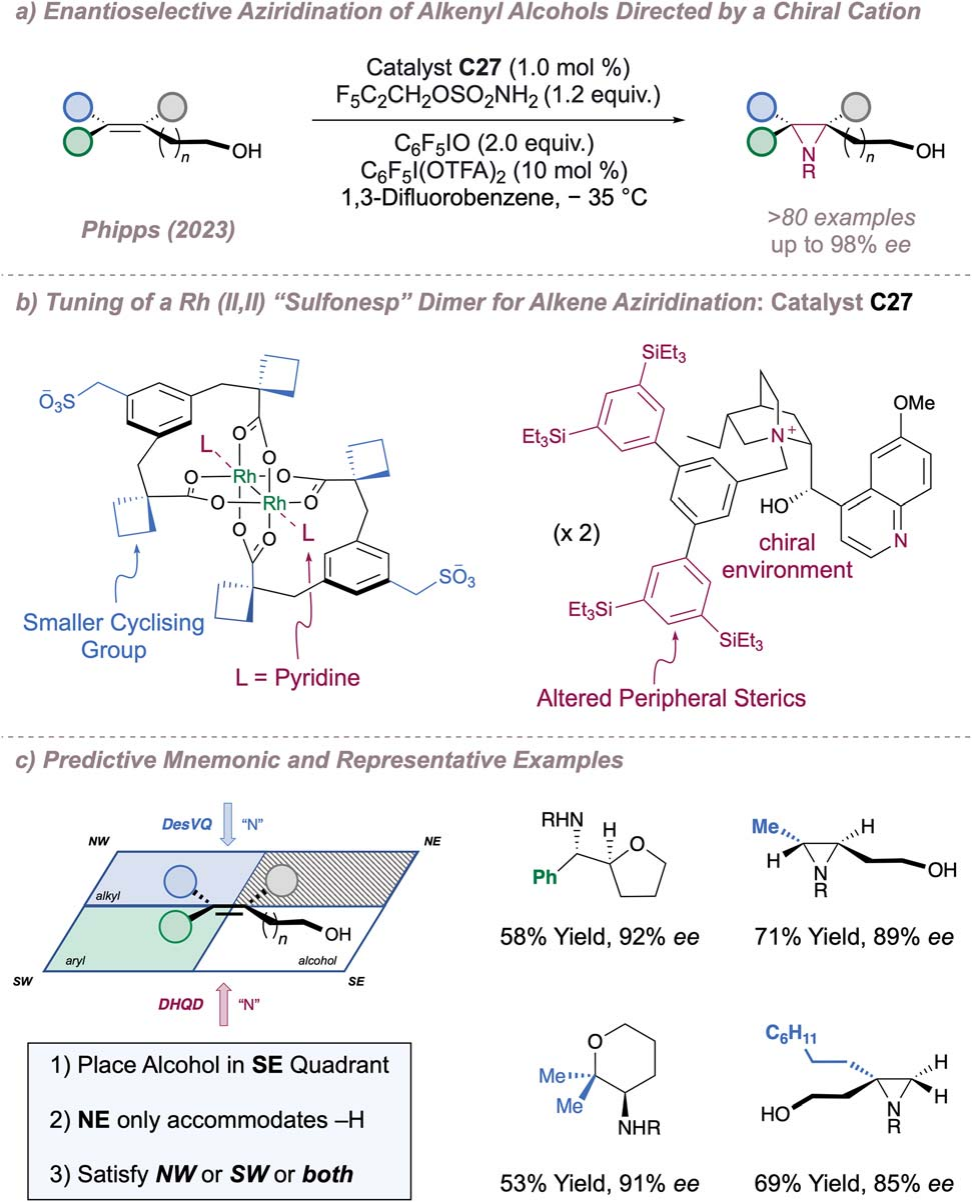
Application of chiral rhodium “Sulfonesp” dimers to the asymmetric aziridination of alkenyl alcohols. In all cases R = –SO_2_OCH_2_C_2_F_5_.

With proof-of-concept in hand, we next questioned whether the catalysts would be capable of differentiating between enantiotopic olefin π faces to perform an asymmetric, substrate-directed intermolecular aziridination of alkenyl alcohols. Reaction optimisation to this end led to the development of a system which was considerably different to the one reported for the previous C(sp^3^)–H amination and which displayed much-improved reaction performance (Fig. 34a and b).^191^ In brief, introduction of the new catalyst C27, in which the axial sites on the Rh dimer are ligated by pyridine (one pyridine ligand per axial site), use of a polyfluorinated oxidant (C_6_F_5_IO) with improved solubility compared to iodosobenzene (C_6_H_5_IO) and addition of a substoichiometric amount of the structurally-related C_6_F_5_I(OTFA)_2_ (which releases trifluoroacetic acid as it is consumed) were all essential to obtain the highest selectivities and yields. These conditions enabled efficient stereocontrolled nitrogen transfer to a range of alkene substitution patterns in moderate to excellent *ee*. Crucially, in addition to the more precedented styrenyl substrates, the reaction also performed well for nitrene transfer to selected unactivated di- and tri-substituted alkenes with both of these substrate classes absent from the prior asymmetric protocols (Fig. 34c, right). In certain cases, intramolecular ring opening of the aziridine by the pendant alcohol was observed or actively promoted and gave rise to enantioenriched aminoetherification products. The data obtained from an extensive substrate scope was used to develop a simple mnemonic based on predominantly attractive interactions between the substrate and the catalyst to predict (i) which olefin substitution patterns are suited to the reaction and (ii) the absolute product stereochemistry obtained using a given chiral cation (Fig. 34c, left). Various post-functionalisation protocols were also developed, particularly focussed on engaging the aziridine and the terminal alcohol group together in order to construct enantioenriched molecules which might be difficult to synthesise by other means.

**Fig. 35:**
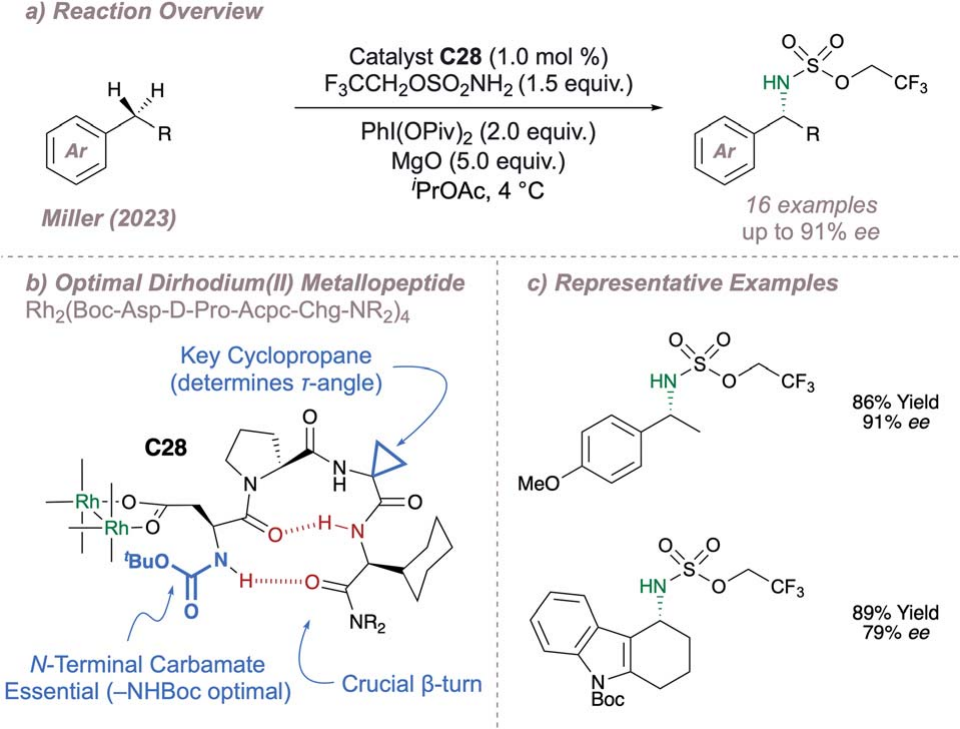
Miller's aspartyl β-turn-based dirhodium(ii,ii) metallopeptide for benzylic C(sp^3^)–H amination.

Continuing with the theme of new chiral Rh(ii,ii) dimer designs, in a very recent publication Miller has disclosed a new catalyst for asymmetric C(sp^3^)–H amination (Fig. 35a and b).^192^ In this design, four β-turn-biased aspartic acid tetrapeptides are used as ligands to span the Rh–Rh core (note that only a single ligand is shown in Fig. 35a for clarity). Such work builds nicely upon Ball's Rh(ii,ii) catalyst designs in which bis(aspartyl) peptide-ligated dimers have been used to perform asymmetric carbene Si–H insertion and cyclopropanation.^183^ A robust ligand exchange protocol ensured facile access to a total of 38 chiral dimers and with such a library the authors were able to present a detailed picture identifying the key ligand motifs required for a successful amination, some of which were rather unexpected. Good enantioselectivities were obtained for amination at a range of benzylic positions (Fig. 35c) and catalyst C28 also proved capable of amination at α-amino C(sp)^3^–H bonds, albeit with no enantioselectivity. Further structural information on these impressive catalysts was acquired through detailed solution and solid-state studies and the wealth of information obtained will be invaluable in the application of these catalysts to ever-more demanding asymmetric nitrene transfer reactions.

## 3. Bridging the gap between synthetic and enzymatic catalysts – metal porphyrins

Transition metal porphyrin [M(Porphyrin)] complexes were amongst the earliest catalysts used to support nitrenes. Inspired by the oxygenase and epoxidase activity of cytochrome P450 enzymes, Breslow^193,194^ and Mansuy^195–197^ in the 1980s independently investigated whether the M(Porphyrin) cofactor alone could catalyse nitrene transfer to saturated C(sp^3^)–H bonds and alkenes. The catalysts were successful for both and these results revitalised the investigation of metal nitrenoid chemistry, which had been initiated by Kwart and Khan some 15 years earlier. However, the first successful examples of enantioselective nitrene transfer using chiral porphyrin complexes were only reported after a further 10 years, by Che.^198–200^

**Fig. 36:**
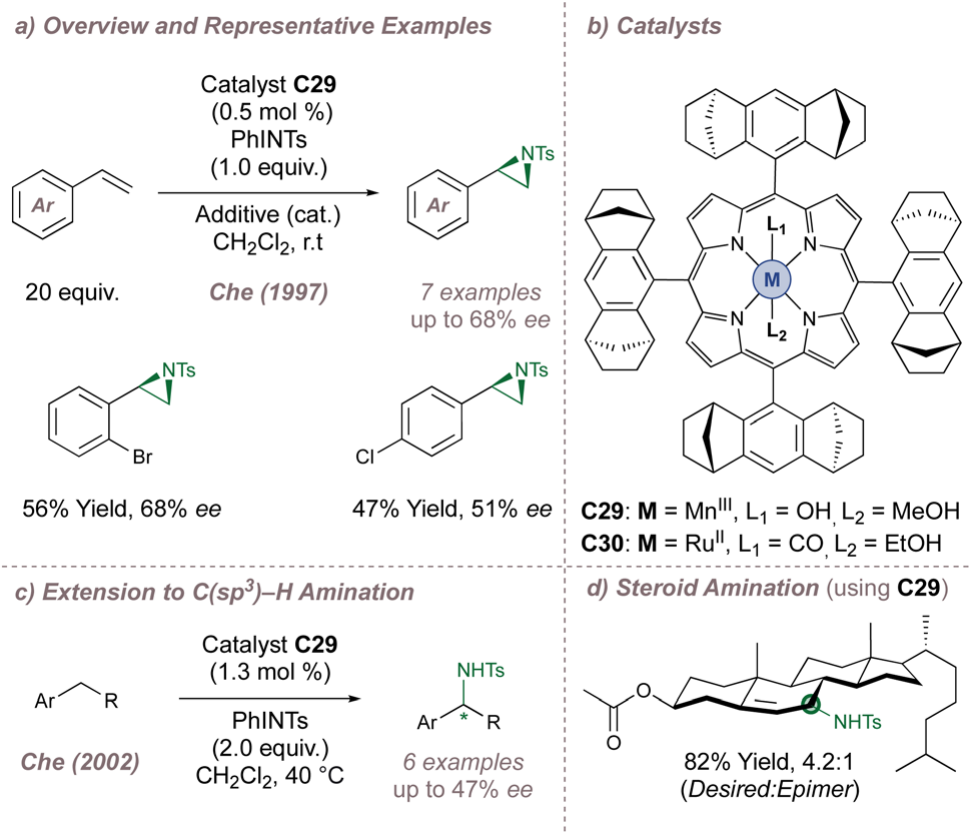
Che's asymmetric aziridination and amination reactions catalysed by chiral M(Porphyrin) complexes. Additive = 4-phenyl-pyridine-*N*-oxide.

In a pioneering example, Che investigated the aziridination of styrene using a catalyst (C29) based on Haltermann's chiral *D*_4_-symmetric ligand which had been previously applied to asymmetric epoxidation (Fig. 36a and b).^198,201^ This porphyrin contains considerable steric bulk arranged at four locations around the ligand periphery and up to 68% *ee* was obtained, with turnover numbers reaching 480. Marchon subsequently reported a similar ligand design, but using peripheral chiral cyclopropyl groups instead.^202^ In the initial report, Che had noted that when posed with a chemoselectivity choice, the chiral catalyst preferred to perform allylic amination over aziridination which initiated studies towards achieving enantioselective amination at activated benzylic positions.^199^ Using the same catalyst, up to 47% *ee* was obtained (Fig. 36c) and an impressive demonstration of catalyst control over regio-, chemo- and diastereoselectivity in the functionalisation of cholesteryl acetate was later demonstrated (Fig. 36d).^200^ The same chiral scaffold was also effective for enantioselective intramolecular amination of sulfamate esters (up to 86% *ee* using C30).^203^ More recently De Bruin reported a similar ring-closing C–H amination of aliphatic azides to construct saturated nitrogen heterocycles.^204^ As above, chiral bulk was appended to the periphery of the porphyrin, although in this case the selectivity peaked at 46% *ee*.

Just as Katsuki significantly advanced the field of M(Salen)-catalysed asymmetric nitrene transfer, Zhang has done the same for Co(Porphyrin) chemistry.^205^ The remainder of this section therefore summarises Zhang's development of a unique family of chiral porphyrin catalysts and their application to various enantioselective nitrogen transfer reactions. Throughout there is a particular emphasis on the use of mechanistic analysis to guide catalyst design.

**Fig. 37:**
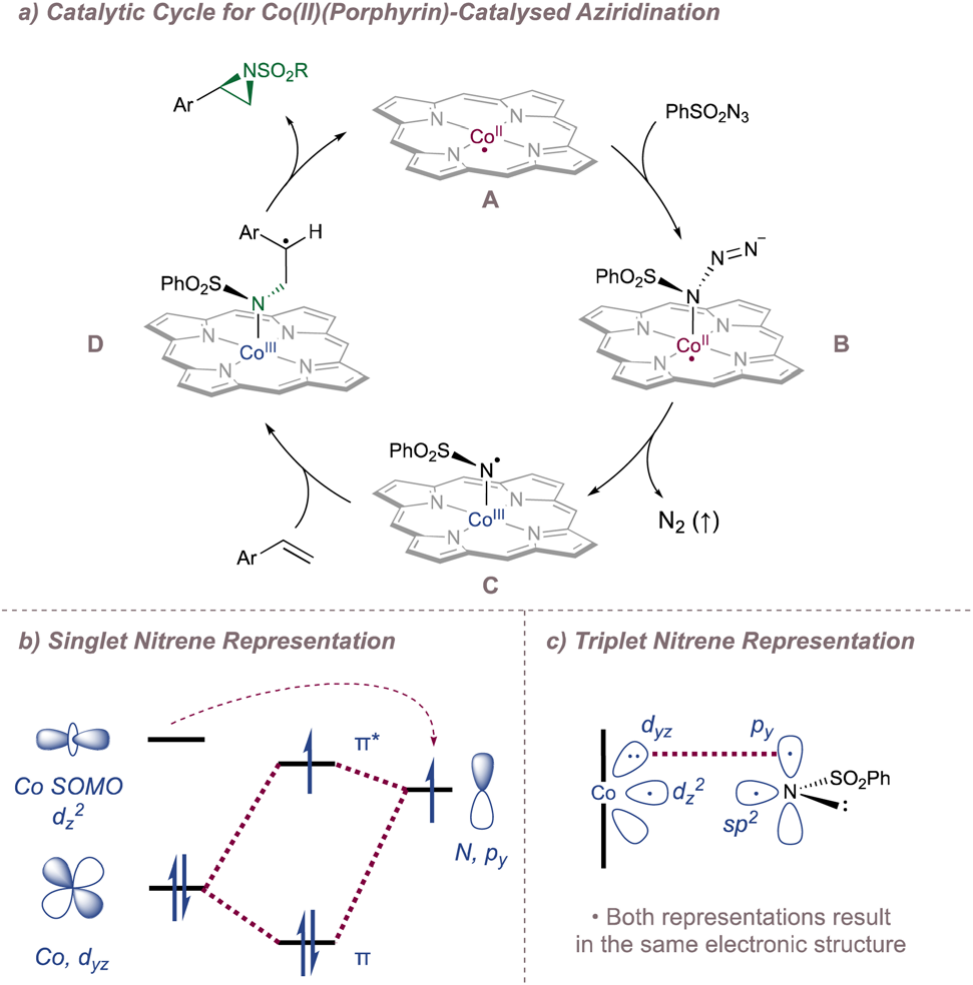
A catalytic cycle for functionalisation using Co(Porphyrin) nitrenoids (upper). Equivalent representations of nitrene radical ligands. Figure adapted from ref. 207 (*Dalton Trans.*, 2011, **40**, 5697–5705).

Before considering questions of selectivity, the mechanism of Co(Porphyrin) aziridination should be discussed as this has implications on enantioinduction, particularly for C(sp^3^)–H amination (Fig. 37).^206^ Reaction of the Co(ii)(Porphyrin) A with a sulfonyl azide forms transient adduct B which affords the nitrene C upon loss of dinitrogen (Fig. 37a). Computations by Zhang, de Bruin and co-workers suggest that C is best described as a one-electron reduced nitrene ligand (or a ‘nitrene radical’ anionic ligand) in which an electron has been transferred from the Co(ii) d_*z*_2__ orbital to the empty p orbital on the nitrogen ligand.^207^ The same electronic structure arises either through a simplified molecular orbital diagram invoking a singlet nitrene (Fig. 37b) or by considering a triplet nitrene (Fig. 37c). Aziridination reactions begin with attack of the *N*-centred radical onto the alkene π-system to form a γ-alkyl radical which then collapses to form the strained three-membered ring (Fig. 37a). The key here is that the system mediates stepwise (as opposed to concerted) nitrogen transfer which is of importance in the work discussed below. The De Bruin group in particular has made key contributions to the understanding of the mechanism of metalloporphyrin aminations and the reader is directed to a number of further studies from that group.^208–212^

**Fig. 38:**
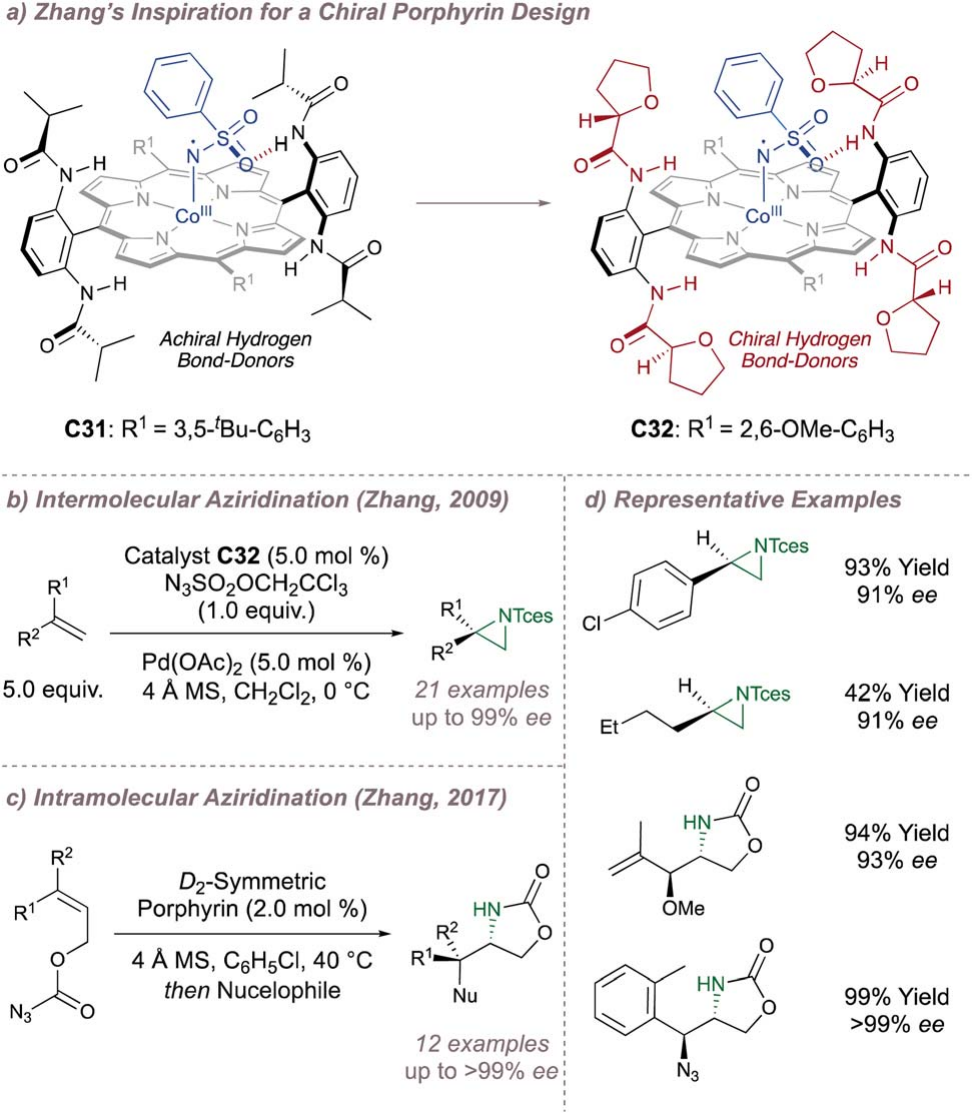
Zhang's development of chiral porphyrin catalysts for enantioselective intramolecular and intermolecular aziridination.

In 2008 Zhang investigated racemic styrene aziridinations using achiral Co(Porphyrin) C31 (Fig. 38a).^213^ A crucial design aspect was the incorporation of hydrogen bond-donating groups on the periphery of the ligand to stabilise the –SO_2_ group on the nitrene radical throughout the catalytic cycle thus significantly enhancing reactivity. Given the close association between the ligand and the nitrene radical throughout the cycle, incorporation of chiral hydrogen bond donors offered the possibility of enantioinduction. This was an exciting proposition since previous work in the field had arguably struggled when applying steric-blocking approaches to porphyrin ligand designs. This is reflected in the poor to moderate enantioselectivities discussed above since the topology of the porphyrin inevitably places the steric bulk at a significant distance from the reactive centre.

In 2009 Zhang reported a family of *D*_2_-symmetric Co(ii) (Porphyrins) with chiral backbone hydrogen bond-donor groups for the asymmetric aziridination of terminal alkenes using TcesN_3_ as the nitrogen source (Fig. 38a and b).^214^ Excellent enantioselectivities were obtained on simple substrates devoid of directing groups and the catalyst C32 could be recycled up to three times without a drop in performance (Fig. 38d, upper). These catalysts were also effective for the enantioselective intramolecular aziridination of allyl azidoformates as was later demonstrated in 2017 (Fig. 38c).^215^ The fused bicyclic aziridines are similar to Schomaker's products described previously (see Fig. 6)^57^ although in this case the increased ring strain and associated instability often required conversion to the 2-oxazolidinones for analysis (Fig. 38d, lower).

**Fig. 39:**
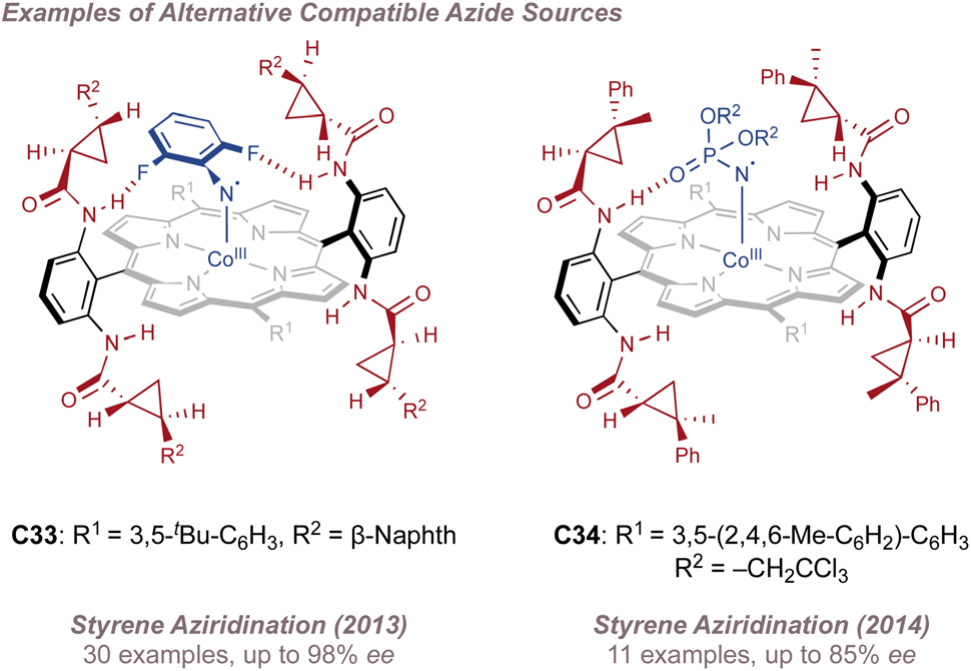
Styrene aziridination using azides containing alternative hydrogen bond-acceptor groups.

Zhang then focussed on intermolecular aziridination using azides bearing alternative hydrogen bond-acceptors. Drawing on the group's extensive catalyst library featuring porphyrins equipped with various chiral hydrogen bond-donors and aromatic ring substituents, other azide sources proved suitable when matched with the correct catalyst. For example, phosphoryl azides gave only moderate results in early studies^216^ but these could be improved through changing the phosphorus substituents and catalyst (C34) together (Fig. 39, right).^217^ Most recently, the carbonyl azide TrocN_3_ was found to give excellent performance and even allowed for the aziridination of electron-deficient alkenes.^218^ Particularly interesting was the use of fluoroaryl azides bearing unconventional hydrogen bond-acceptors (Fig. 39, left). The direct synthesis of *N*-aryl aziridines from anilines or their derivatives^219^ has the potential to replace lengthy aziridination/deprotection/*N*-arylation sequences but methods to achieve this are under-represented. Zhang hypothesised that an *ortho*-fluorine atom on an aryl azide could act as the hydrogen bond acceptor leading to efficient catalyst recognition for this type of nitrogen source also. Using catalyst C33, a variety of terminal styrenes were transformed to the corresponding *N*-fluoroarylaziridines.^220^ Whereas a single *ortho*-fluorine atom could induce reasonable *ee* (75% *ee*), better results were obtained with double *ortho* substitution (96% *ee*). In the absence of *ortho*-fluorine atoms no reaction was observed, even with fluorination at several other positions on the ring. The same azides were later shown to be competent for asymmetric benzylic C(sp^3^)–H amination.^221^

**Fig. 40:**
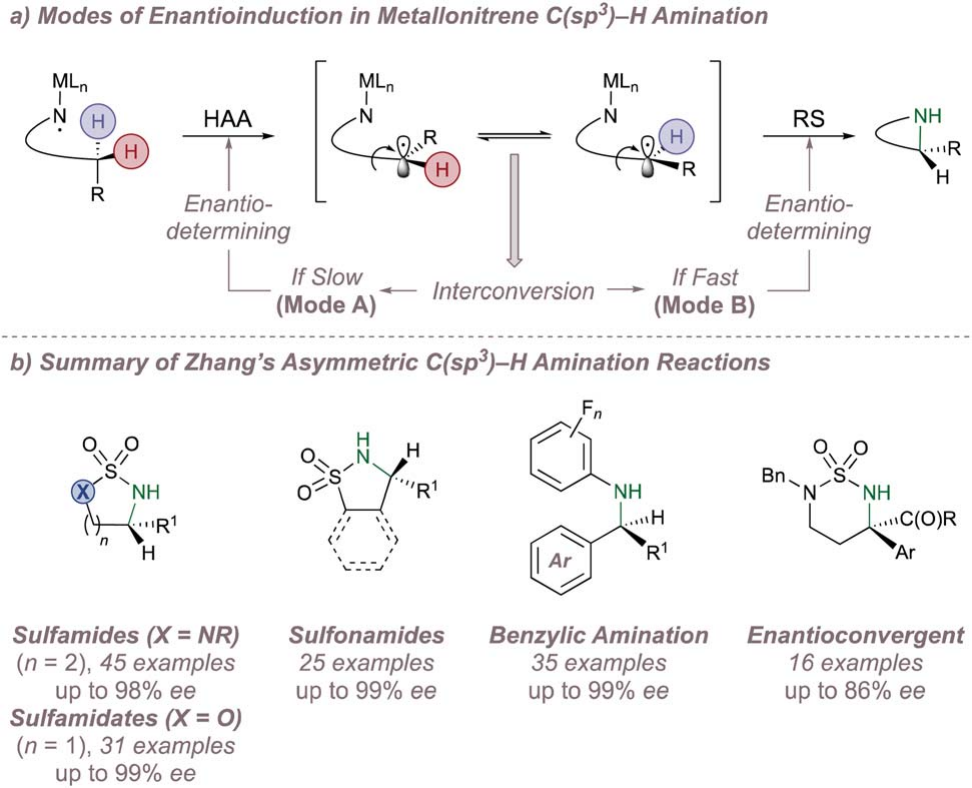
Enantioinduction modes in asymmetric metallonitrene amination.

These *D*_2_-symmetric catalysts have also been applied to several other C(sp^3^)–H amination reactions. Before proceeding, it is important to recognise how the mechanism of the C–H functionalisation can affect enantioselectivity (Fig. 40a). Taking an intramolecular reaction as an example, as with aziridination there are two stages to the stepwise process. In the first, (1, *n*) intramolecular hydrogen atom abstraction (HAA) gives an alkyl radical which is followed by a radical substitution (RS) step in the second during which the C–N bond is formed and the catalyst regenerated. If the alkyl radical is capable of rapid interconversion prior to RS, then RS is enantiodetermining (mode B). However, if interconversion is slow then the HAA is enantiodetermining (mode A). The *D*_2_-symmetric catalysts can mediate either mode depending on reaction conditions and catalyst design. A summary of Zhang's (predominantly intramolecular) asymmetric C(sp^3^)–H aminations developed up until 2022 are shown in Fig. 40b and some selected examples are now discussed in greater detail.

The first of the group's C(sp^3^)–H aminations reported the intramolecular 1,6-amination of sulfamoyl azides to form sulfamides (Fig. 40b).^222^ The initial HAA was feasible at non-activated and even deactivated C(sp^3^)–H bonds and was highly 1,6- selective even if this resulted in the homolytic cleavage of stronger C–H bonds in the presence of weaker ones. Changes in regioselectivity were observed when moving to different azide sources, resulting in the formation of 5-membered sulfonamides with aryl or alkylsulfonyl azides^223^ and strained 5-membered sulfamidates^224^ when using alkoxysulfonyl azides (Fig. 40b). The sulfamidates are particularly prone to stereospecific nucleophilic ring-opening at the carbon atom adjacent to oxygen and can be considered as latent β-functionalised chiral amines. Mechanistically, these transformations are all thought to occur *via* mode B.

**Fig. 41:**
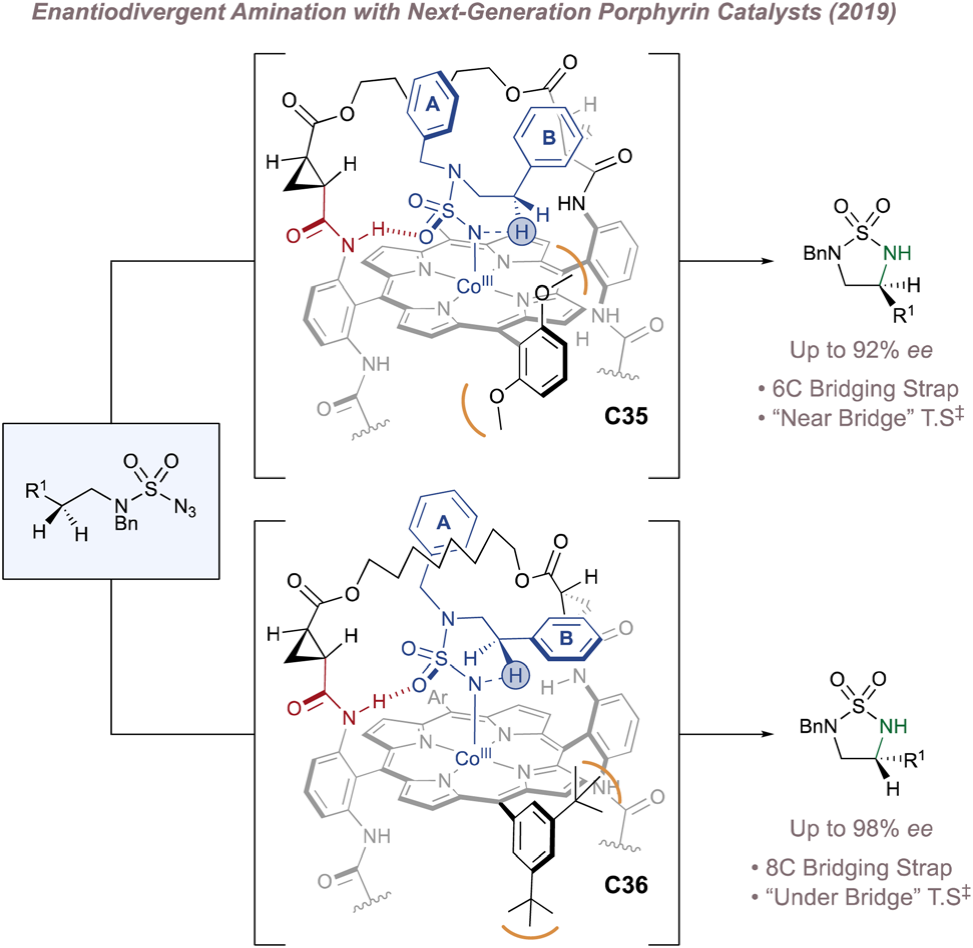
Catalyst design leads to enantiodivergent amination, even with the same chiral hydrogen bond-donors. For each transition state only the top half of the porphyrin is shown for clarity.

Zhang reported an exciting advance in ligand design in 2019 which involved internal bridging of the chiral amide groups with alkyl chains of varying lengths (Fig. 41).^225^ The development of this ligand class has had significant impact on enantioselective amination because in these next-generation catalysts, the open chiral pocket is now converted into a closed chiral cavity. The resultant increased rigidity improves the hydrogen bond-donating ability of the amides and the volume of the cavity can be adjusted by changing the alkyl strap length. In catalyst validation studies these next-generation porphyrins outperformed the previous designs on benchmark aziridination and cyclopropanation reactions but more exciting were the new modes of enantiocontrol afforded by the novel designs.^225^

An example is the enantiodivergent intramolecular 1,5-amination of sulfamoyl azides (Fig. 41), reported by Zhang later in 2019.^226^ The report is intriguing because equal and opposite enantioselectivities were obtained using catalysts C35 and C36 which contain identical chiral amide motifs and differ only in seemingly inconsequential peripheral elements of the scaffold (the length of the alkyl straps and the identity of the peripheral aromatic rings). This surprising result was rationalised using in-depth mechanistic and DFT studies. In this case, radical interconversion following the HAA is slow due to the confines of the chiral cavity hindering rotation about the C–C bond. Since the second RS step is rapid, this locks in the stereochemistry from the HAA which is therefore enantiodetermining (Fig. 40a, mode A). For catalyst C35 the favoured transition state for HAA features aryl ring A near the bridge, with ring B minimising clash with the 2,6-OMe group thus positioning the pro-(*S*) benzylic bond for abstraction. By contrast the longer strap length (and hence larger cavity) for C36 allows ring A to slip under the bridge with the B ring once again minimising clash, this time with the peripheral 3,5-^*t*^Bu–Ph. This now correctly positions the pro-(*R*) benzylic bond for abstraction. As before, hydrogen bonding with the peripheral amide was essential for transition state organisation in both cases.

In order to lend support to the intricate models for enantioinduction derived from the calculations, substrates with bespoke electronic or steric properties were subjected to the reaction conditions to probe the interactions that would arise between these and the catalysts in the proposed transition states. In terms of electronics, substrates bearing heteroatoms (ester, furan or thiophene) close to the site of C(sp^3^)–H amination led to poor reactivity and selectivity outcomes as a result of lone-pair repulsion operating between these and the 2,6-OMe group in C35. By contrast, no such electronic interactions would be expected with the 3,5-^*t*^Bu groups in C36 and indeed in those cases the products were formed in excellent yield and *ee*. Considering sterics, C35 significantly outperformed C36 in the amination of substrates bearing particularly bulky groups (such as a ferrocene). This is entirely consistent with the sterically-demanding group being oriented out of the catalyst cavity and away from the porphyrin ring in the transition state (Fig. 41, top) and hence not significantly impacting the *ee*.

**Fig. 42:**
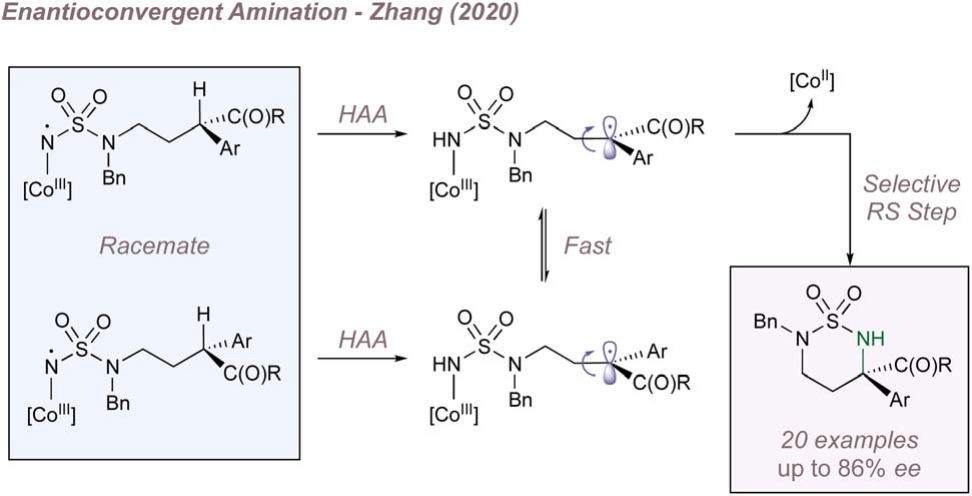
Enantioconvergent C–H amination catalysed by a chiral porphyrin leading to α-tertiary amine stereocentres.

Within the context of enantioconvergent reactions, the advantage of radical *versus* concerted C(sp^3^)–H insertion is that racemic starting materials can in principle be converted to a single amine product, providing that interconversion between the alkyl radicals is fast enough (Fig. 40a, mode B) to racemise the stereocentre. This was demonstrated by Zhang in 2020 with the enantioconvergent intramolecular amination of racemic alkylsulfamoyl azides.^227^ Referring to Fig. 42, in this instance the RS step is enantiodetermining and requires careful tuning of the constraints of the catalyst pocket. Enough space is needed to ensure effective alkyl radical interconversion but the pocket must be sufficiently discriminating for selectivity to be achieved in the rebound step. A first-generation Zhang porphyrin fulfilled these criteria and was particularly effective at discriminating between aromatic and ester groups in the RS step thus enabling the construction of protected and enantioenriched α-chiral tertiary amines. There was an unusual increase in *ee* with increasing temperatures up to 50 °C, later identified as the optimum temperature for accelerating radical interconversion without eroding selectivity in the subsequent RS. In addition, KIE data proved that there was a modest preference for HAA from one of the starting material enantiomers over the other (in a matched *vs.* mismatched scenario) but this was countered by almost complete racemisation of the alkyl radical prior to recombination meaning that C–N bond formation was the enantiodetermining step. This was confirmed experimentally when two enantiomers were each subjected separately to the reaction conditions using the same catalyst. In both cases the reactions converged to the same enantioenriched product with identical *ee*. Overall, this transformation was a long-standing objective of the Zhang group since all previous intramolecular aminations had only ever formed secondary carbon stereocentres.

**Fig. 43:**
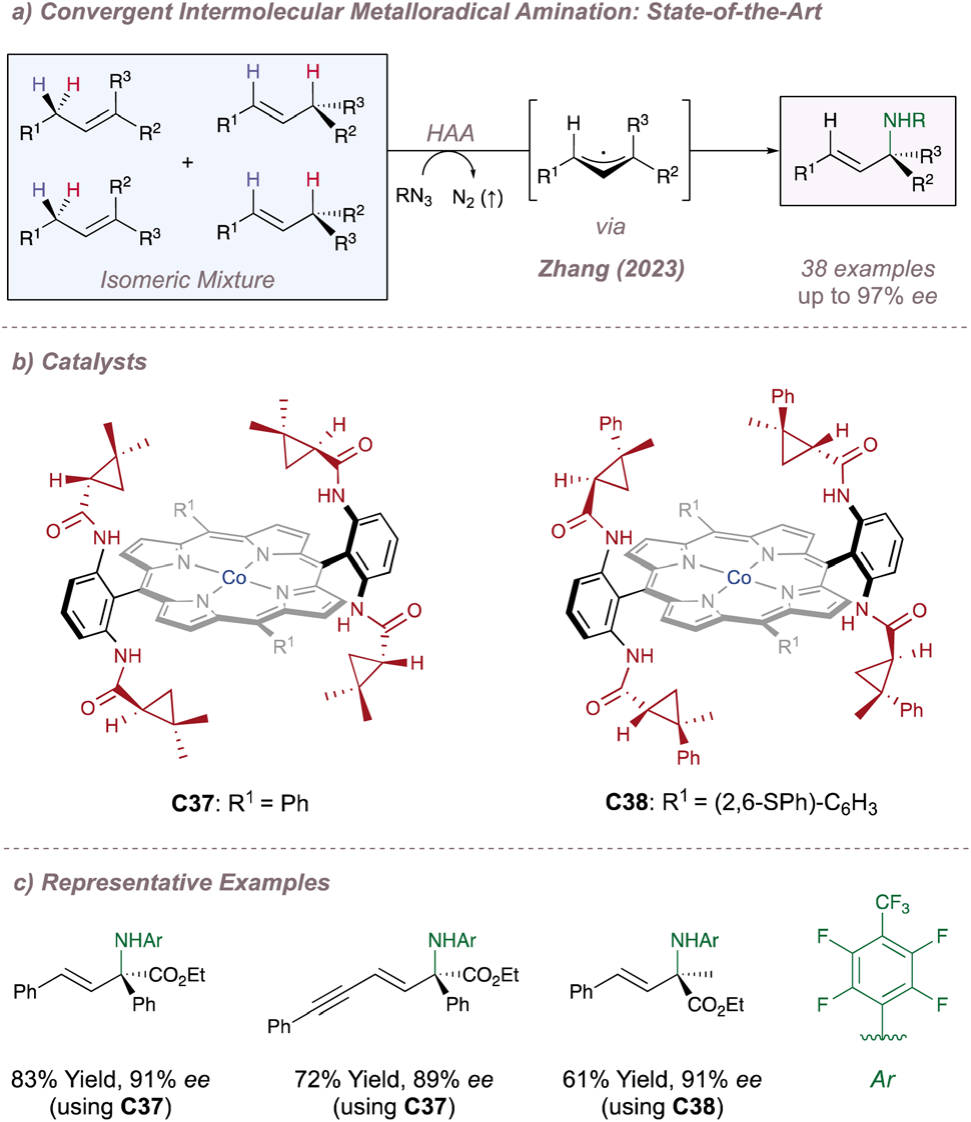
Highly convergent metalloradical-catalysed allylic amination.

In the most recent report, Zhang disclosed a highly convergent amination reaction using Co(ii)-based chiral metalloradical catalysis to form α-tertiary chiral allylic amines (Fig. 43).^228^ The reaction begins with the familiar HAA, this time from an allylic position followed by the RS step. However, in this case, simultaneous control over four aspects of selectivity is required to ensure a successful outcome. Fundamental to this is the catalyst's role in determining the fate of the equilibrating intermediate allylic radical which is formed within the confines of the active site (Fig. 43a). First, the catalyst must control whether the secondary or tertiary end of the allyl radical undergoes RS (regiocontrol). Second, the catalyst must control the absolute configuration of the aminated proximal chiral stereocentre (enantiocontrol) as well as determining the distal alkene double bond geometry (diastereocontrol). Before even considering these challenges, there is also the broader question of chemoselectivity in which the catalyst must first decide between performing C–H amination or aziridination. Remarkably, the first-generation Zhang porphyrin C37 and the second-generation C38 were capable of effectively controlling all four aspects thanks to a finely-balanced network of attractive non-covalent interactions operating between all three of the catalyst, the nitrogen source and the substrate (Fig. 43b). Tellingly, when the control Co(TPP) catalyst was used, complex product mixtures were observed in the same instances where C37 and C38 delivered single aminated products. A unique implication of the high convergence of the process is that mixtures of isomeric alkene starting materials can be used in the reaction, and then funnelled to a single aminated product. This is unusual yet very attractive given that it is often difficult to obtain single isomers of such complex alkene starting materials. In addition to reporting an ample scope with respect to the substitution of the allyl starting material (Fig. 43c), the authors demonstrated the viability of other azide sources alongside showcasing the synthetic potential of the aminated products through several post-functionalisation protocols. Overall, this report sets a gold standard for the control of selectivity in radical reactions and is a testament to the effectiveness of attractive non-covalent interactions in influencing multiple selectivity outcomes in challenging asymmetric transformations.^229^ Along with Chang's copper-catalysed example discussed above, this work highlights the unique potential of metalloradical amination reactions in converting starting material mixtures to single, valuable products. The outcome is intimately linked to reaction mechanism and offers new synthetic opportunities that are not possible when using the concerted C(sp^3^)–H aminations explored throughout this review.

We note that Zhang has also applied these catalysts to the analogous intra- and intermolecular carbene-transfer processes with equally impressive successes. These lie outside of the scope of this work but the interested reader is directed to a recent review.^230^

**Fig. 44:**
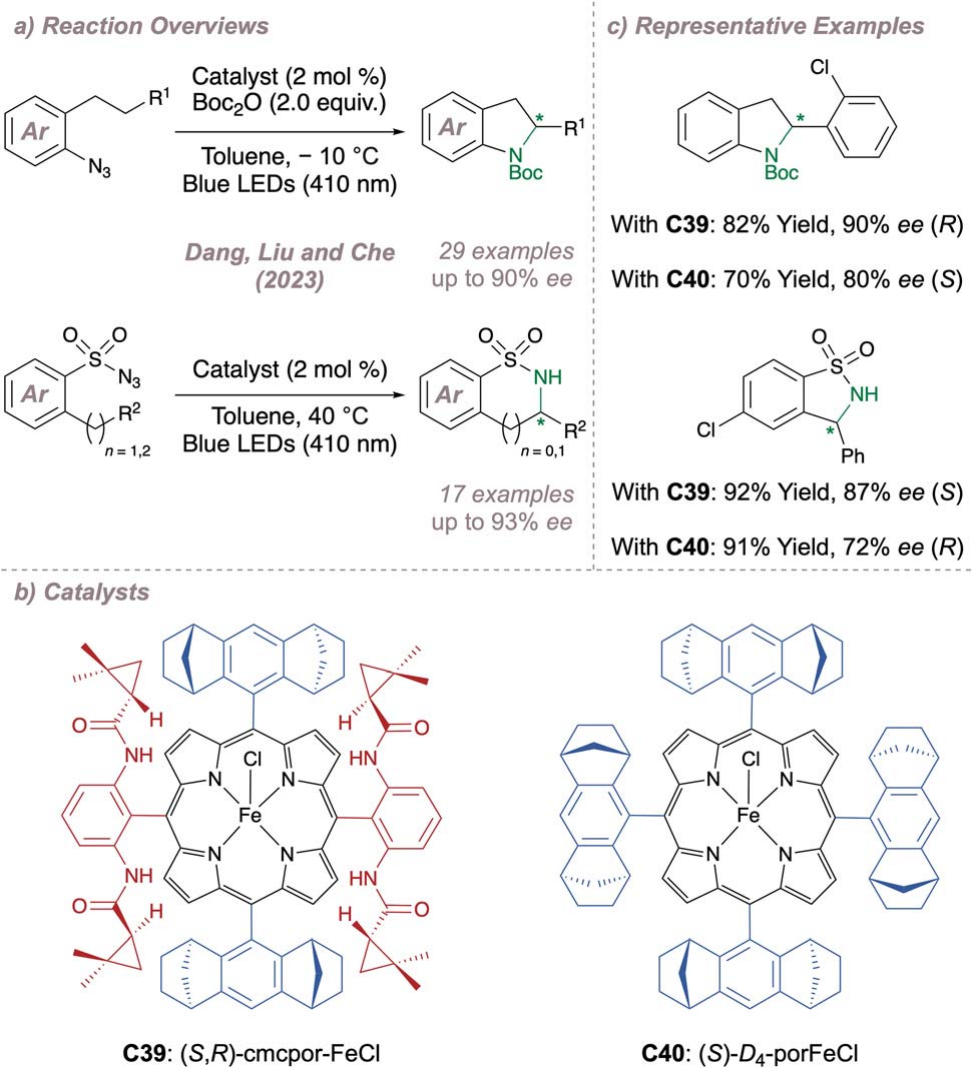
Application of a new chiral porphyrin design to enantioselective intramolecular nitrene cyclisations. The absolute stereochemistry of the new chiral centre is denoted in brackets.

The final chiral porphyrin catalyst class to consider has been reported very recently by Dang, Liu and Che (Fig. 44b).^231^ This novel and intriguing design combines the steric-blocking elements (in blue) from the *D*_4_-symmetric ligand discussed at the beginning of this section along with the hydrogen bond-donating capability (in red) of Zhang's *D*_2_-symmetric ligands. It was hypothesised that by combining both of these elements into one catalyst, a more selective nitrenoid transfer might be possible. Based on this idea, the synthesis of a number of such porphyrins was reported and it was found that the (*S*,*R*)-cmcporFeCl catalyst C39 was optimal in mediating the enantioselective cyclisation of aryl azides and arylsulfonyl azides, using blue LED activation under mild conditions (Fig. 44a). As a control, the *D*_4_-symmetric catalyst (*S*)-*D*_4_-porFeCl C40 which only contains the steric-blocking elements was tested alongside C39 in both reactions. In the cyclisation of the aryl azides, the selectivities and yields obtained with both were comparable, albeit with the opposite absolute stereochemistry in the products. However, for the arylsulfonyl azide cyclisation, C39 typically gave better and opposite selectivity compared to C40 (Fig. 44c). It was important to correctly match the absolute configuration of the blocking elements with that of the chiral amides and in catalyst C39 these work synergistically to afford products with moderate to good *ee*. If however, the stereochemistry on the blocking elements is inverted while keeping the absolute stereochemistry of the amides the same, the selectivities are much reduced. DFT calculations revealed that the reaction proceeds through a HAT/rebound mechanism and the intermediacy of a carbon-based radical was demonstrated experimentally not only through EPR studies but through the inhibition of product formation when using the radical scavengers TEMPO and BHT. Further both attractive non-covalent interactions and steric repulsion effects operating between the substrate and the ligand were found to be responsible for the high selectivities, as hypothesised. With the design of C39 now validated, it will be interesting to see if it is effective also for intermolecular reactions.

## 4. Enzymatic catalysis

In this final section we examine contemporary enzymatic methods for nitrene transfer. We leave this analysis until last because we wish to contrast the following enzymatic methods, not only with the clearly-related porphyrin catalysts in the preceding section, but with small molecule catalysts as a whole. In doing so we hope to convey a sense of just how rapid development in this sub-field has been, since the performance of these systems in some cases can now match or indeed surpass the state-of-the-art small molecule methods and there are exciting early indications that the enzymatic approach may be able to tackle outstanding challenges such as site- and enantio-selective functionalisation of unactivated positions.

**Fig. 45:**
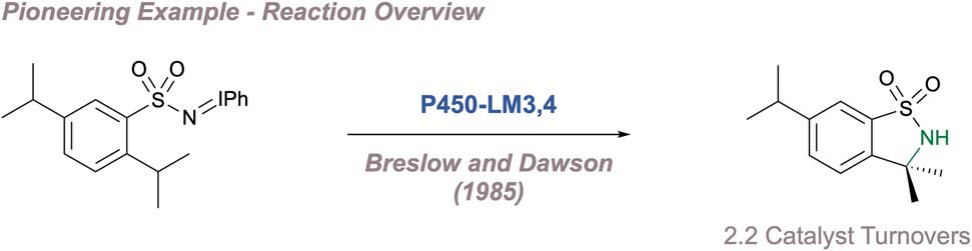
Breslow and Dawson's initial application of a CYP to asymmetric intramolecular nitrene transfer.

Building on Kwart and Khan's initial study of copper nitrenoids,^10,11^ Breslow^193,194^ and Mansuy's^195–197^ early investigations using M(Porphyrin) complexes for nitrene transfer were a key advance in the field. Given that this work had the goal of translating the oxygenase activity of Cytochrome P450s (CYPs) to nitrogen-transfer using the porphyrin co-factor alone, the next step was to try and use a whole enzyme to perform the same reaction. This was indeed reported in 1985 by Breslow who demonstrated that rabbit P450-LM3,4 could catalyse the intramolecular C(sp^3^)–H amination of an iminoiodinane with a turnover number of 2.2 (Fig. 45).^232^ Although enzymatic turnover was low (likely due to the competing hydrolysis of both the substrate and the iron-nitrenoid under aqueous conditions). Arnold notes that this example is important for two reasons.^233^ First, it shows that enzymes can catalyse reactions for which they were not seemingly designed by nature and second that it might be possible to take a reaction developed in the laboratory and then refine an enzyme to catalyse this “new-to-nature” transformation. On this second point, often as chemists we draw inspiration from nature in biomimetic approaches to synthesis but there are some reactions developed in laboratories that to the best of our current knowledge have no natural counterpart. However, this does not mean that enzymes will be unable to catalyse these. Biology is a rapid learner and encouragement in the form of an applied selection pressure is sufficient to begin directed enzymatic evolution towards a new transformation.^234^

Key to achieving the second point is the concept of enzyme promiscuity.^235^ Although enzymes are exceptional catalysts for a specific transformation they can sometimes catalyse other reactions to a degree in the first instance. A glimmer of activity for another reaction is sufficient to start a program of directed evolution for the enzyme in which “fitness peaks” are scaled by mutating a single amino acid residue at a time.^233,236–238^

**Fig. 46:**
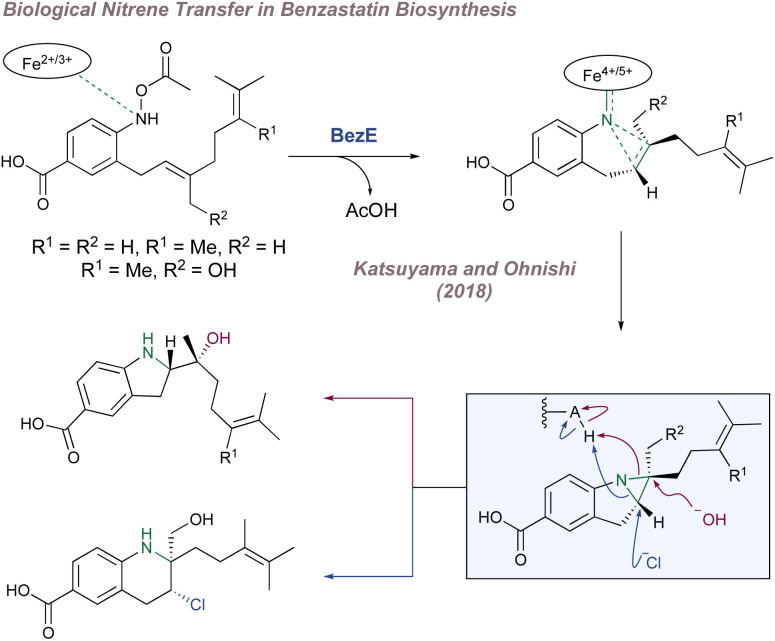
A rare example of biological nitrene transfer which was discovered after the directed evolution of enzymes to perform the same reactivity.

Arnold identified CYPs as excellent candidates for directed evolution precisely because of their promiscuity. In particular, P450_BM3_ from *Bacillus megaterium*^239^ has served as the common starting point in the directed evolution of enzymes for carbene transfer (to alkenes, alkynes, Si–H, B–H and C–H bonds) and nitrene transfer.^238^ The outstanding activity of CYPs for hydroxylation in nature has been well documented^240^ but formation of carbon–nitrogen bonds by an analogous mechanism is far rarer. Instead, nature generally prefers to introduce C(sp^3^)–N linkages through processes in which nucleophilic amines attack electrophilic carbonyls or alkenes.^241–245^ Although biological C–N bond formation using metal nitrenoids is practically unknown at present (see Fig. 46 for a notable exception) this does not necessarily mean that enzymes are inherently incapable of mediating this reactivity. In the following section a summary of asymmetric enzymatic nitrene transfer chemistry developed through directed evolution is presented and highlights how evermore challenging aminations have been realised over time with excellent control over all aspects of selectivity.

**Fig. 47:**
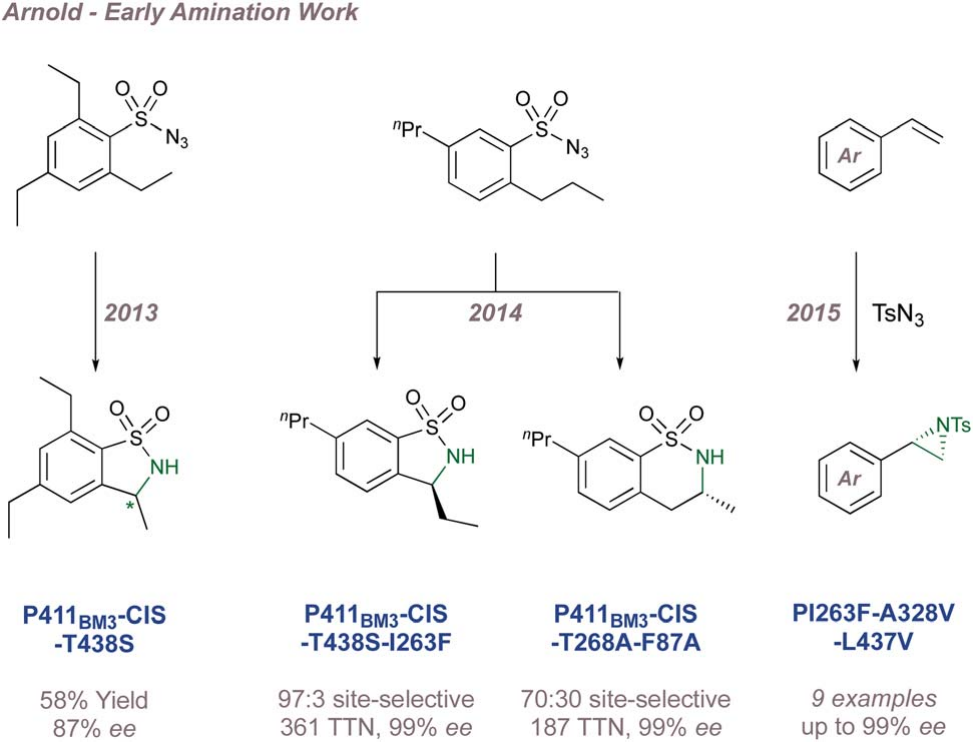
Early CYP-catalysed nitrene transfer reactions developed in the Arnold laboratory. TTN = total turnover number.

Inspired by Breslow's initial intramolecular amination, in 2013 Arnold examined the same reaction using a sulfonyl azide in place of the iminoiodinane due to the much improved aqueous solubility and stability of the former.^246^ Ultimately, formation of the sultam was catalysed in cells expressing P411_BM3_-CIS-T438S and the product could be isolated in 58% yield and 87% *ee* (Fig. 47, left).^246^ It is worth noting here that P411 CYPs differ from P450s in that the iron-coordinating cysteine is replaced by a serine with a concomitant change in the Fe(iii)/Fe(ii) reduction potential. Fasan later reported a very similar system obtaining up to 91% *ee*^247^ and in 2017 Hartwig disclosed a complementary approach, again with a very similar substrate but with replacement of the heme with an (Ir(Me)-PIX) cofactor.^248^ In 2014 Arnold examined another system and reported an enzyme which gave improved enantioselectivity for cyclisation to the 5-memebred sultam (P411_BM3_-CIS-T438S-I263F) alongside a new variant (P411_BM3_-CIS-T268A-F87A) engineered to catalyse formation of the 6-membered heterocycle from the same starting material and with good regioselectivity and excellent enantioselectivity (Fig. 47, centre).^249^ This is an outstanding example of catalyst control and compares very favourably to the performance of small molecule catalysts developed for the same transformation (see Fig. 16a).^101^

**Fig. 48:**
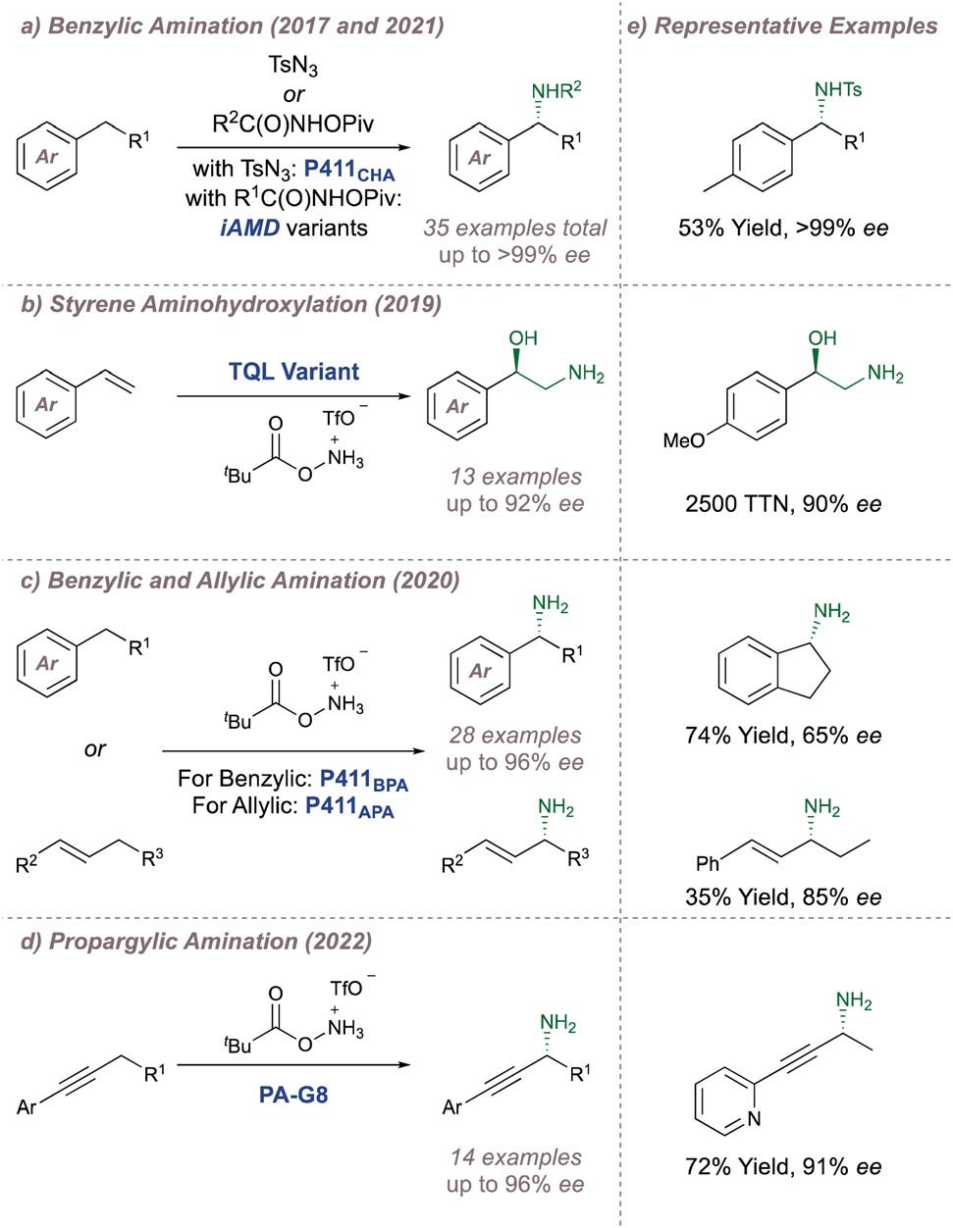
More challenging intermolecular CYP-catalysed nitrene transfer reactions developed in the Arnold laboratory. TTN = total turnover number.

Further examples of C(sp^3^)–H amination have been reported by the same laboratory including protocols for asymmetric intermolecular benzylic amination (Fig. 48a),^250,251^ and asymmetric intramolecular amination at secondary and tertiary C–H bonds.^252^ Parallel to these studies, Arnold has also investigated the asymmetric aziridination of styrenes, with an initial report in 2015 (Fig. 47, right).^253^ What is most interesting is that this last reaction predates the discovery of the first known cytochrome P450 nitrene transferase. Only in 2018 did Katsuyama and Ohnishi report that the BezE enzyme, catalyses a key intramolecular aziridination using an iron nitrenoid during benzastatin biosynthesis (Fig. 46).^254^

One drawback common to all of the asymmetric nitrene transfer reactions discussed in this review is the requirement for a protecting group on the nitrogen which adds extra (often non-trivial) steps to reveal the free amine. Arnold recognised this and has moved to using hydroxylamine-derived nitrene precursors which transfer –NH_2_ directly following activation at the metal.^255^ These reactions are thought to proceed *via* an Fe=NH species which is the closest nitrogen analogue to the natural Fe=O intermediate involved in biological C–H hydroxylation.^240^ This was first demonstrated in the asymmetric aziridination/ring opening reaction of styrene catalysed by a *Rma* cyt c “TQL” (Fig. 48b).^256^ The same nitrene source was then applied to intermolecular C–H amination at benzylic and allylic positions using P411 variants^257^ (Fig. 48c) and now more recently to propargylic positions using PA-G8 (Fig. 48d).^258^

It is important to consider the trajectory of these engineered enzymes for the purposes of asymmetric nitrene transfer. Arnold's first asymmetric amination using an engineered CYP was reported in 2013 on a simple substrate. Already, only 10 years later, the methodology can catalyse asymmetric intermolecular amination at most classes of activated C(sp^3^)–H bond, with the highest selectivities, and can often deliver the free amine directly, something which no other current asymmetric nitrene transfer protocol achieves. The progression has been meteoric and underscores the power of this approach.

**Fig. 49:**
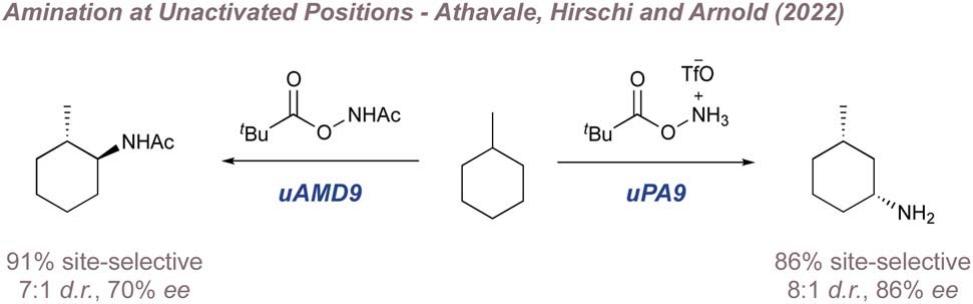
Initial results towards the selective functionalisation of unactivated hydrocarbons using engineered CYPs.

The last frontier for these enzymes and for asymmetric intermolecular amination in general is the selective functionalisation of completely unactivated aliphatic hydrocarbon positions which is the ultimate aim of C–H functionalisation.^259^ Studies in the Hirschi and Arnold laboratories have recently focussed on this challenge by examining the intermolecular amination of feedstock alkanes such as alkyl cyclohexanes (Fig. 49).^260^ Encouraging initial results have been disclosed wherein methyl and ethyl cyclohexane can be aminated or amidated with control of site-selectivity, diastereoselectivity and enantioselectivity. The corresponding reactions on other cyclic and linear substrates show generally decreased selectivities and yields for the moment. It will be a triumph for directed evolution if intermolecular hydroxylation can be mirrored with aminations on the very same unactivated substrate classes and with identical precision. Given the pace at which this field is advancing, this seems to be a matter of when rather than if.

## 5. Conclusions and outlook

In conclusion, this review has aimed to provide a detailed overview of the various strategies used to impart enantioselectivity for metal nitrenoid insertion into C(sp^3^)–H bonds and addition to alkenes. Although there are features common to many of the substrates employed in the various sections, the design tactics used to control enantioselectivity are unique to each catalyst class and required creativity and an understanding of reaction mechanism to achieve effective enantioinduction. A variety of ligand classes for transition metals have been gradually tuned and developed to become highly effective in a range of scenarios and in recent years these have been joined by enzymatic protocols which are already providing competition to the more classical methods. The rate of development of enzymatic methods, in particular, demonstrates great promise in terms of addressing future challenges in the area, which will be outlined below, considering C–H amination and aziridination in turn.

For C–H amination, functionalisation at activated positions such as benzylic, allylic or propargylic C–H bonds is dominant and in most cases the reactivity of the metal complexes cannot be extended to less activated C–H bonds. Yet this is a crucial goal if the methodology is to enjoy more widespread uptake. The successful realisation of this aim will require the design of catalysts which are both more reactive (capable of functionalizing sites of lower electron density) and more discriminating (capable of functionalizing sites with minimal steric bias such as specific methylene positions in the middle of a long alkyl chain). The challenge of site-selectivity is one which is not the focus of this review. However, as more reactive nitrene transfer catalysts are inevitably developed that are able to react with unactivated secondary C(sp^3^)–H bonds, the issue of site-selectivity will become paramount. Importantly, this will need to be addressed alongside enantioselectivity since, in most cases, new stereocentres will be formed in such processes. This triple challenge of higher reactivity, site-selectivity and enantioselectivity will certainly stretch current chemical catalysis concepts to the limit and it could be argued that the traditional approach of the metal complex bearing a chiral ligand may struggle to achieve all three aspects simultaneously.

For aziridination there remain limitations regarding the compatible substrate classes, with styrenes being arguably over-represented in existing advances and application to unactivated alkenes being much rarer. Compared with asymmetric epoxidation, one of the most widely used catalytic asymmetric reaction types, asymmetric aziridination is far less employed by practicing synthetic chemists. This is despite the fact that nitrogen atoms are as similarly prevalent in chiral compounds of medicinal and societal relevance as oxygen atoms. The reason is the current gap in terms of methodology and the breadth of substrates that can be tackled. There is little doubt in these authors' minds that if highly effective and general ‘aza’ versions of the Sharpless asymmetric epoxidation or dihydroxylation were developed then they would be rapidly adopted by the synthetic community.

Given the above challenges that remain, what approaches might allow these to be tackled?

Notably, the exploitation of attractive non-covalent interactions between designed ligands and substrates has increasingly been identified as impacting on enantioselectivity outcomes. In many cases highlighted in this article the importance of non-covalent interactions has been realised retrospectively through DFT analysis. This is important because it demonstrates that steric repulsion is not the only force at play and sometimes bulky groups can actually be engaging in unexpected attractive interactions, such as in the case of London dispersion forces. But there are increasingly examples where attractive non-covalent interactions have been incorporated deliberately as part of an explicit design strategy. In the latter case, such an approach can offer alternative ways of incorporating asymmetry into a transition metal complex compared with the conventional incorporation of stereocentres covalently into the optimal ligand scaffold for an achiral reaction. It is certain that the latter approach has many more innovations to come but it is also evident that designed approaches harnessing non-covalent interactions are gaining traction and are likely to continue to do so in the coming years as confidence in their deployment increases. We additionally believe that further development in the use of designed non-covalent approaches will be key to addressing the site-selectivity challenges that will also be invariably associated with more challenging reactivity scenarios such as remote functionalisation, away from an arene or alkene (*vide supra*).

Considering the collection of catalyst classes presented above, it seems that engineered enzymes hold remarkable promise. The next decade or so will likely show whether enzymatic approaches overtake purely synthetic catalysts to become the system of choice for performing the most challenging and currently elusive aminations. As previously pointed out in this review, within only a decade or so, engineered P450 enzymes have already matched the selectivities obtained with small-molecule catalysts in a number of ‘benchmark’ substrate types. Furthermore, they have made important progress towards the asymmetric intermolecular transfer of (i) unprotected nitrogen atoms at (ii) unactivated positions. Combined, the latter two points comprise an important challenge in contemporary nitrene transfer to which small-molecule catalysts have arguably not yet risen. This is clear through a consideration of the examples included above in which nitrogen protecting groups abound and in which despite the breadth of catalyst structures and strategies considered, it is consistently the same benzylic and allylic positions which are being targeted. As a result, there are currently a very large number of synthetic catalysts to tackle asymmetric amination of essentially rather similar substrates. A caveat here is that the turnover numbers and predictability in these pioneering enzymatic efforts require much improvement for them to be used as genuine synthetic tools. Whilst the groundwork has been laid on which these developments can occur on, their translation to the everyday toolkit of organic chemists is still some distance away and it will be fascinating to reassess this situation in a decade's time.

## Author contributions

The manuscript was written through the contributions of all authors and all authors have given approval to the final version.

## Conflicts of interest

There are no conflicts to declare.

## Supplementary Material
